# Automated color detection in orchids using color labels and deep learning

**DOI:** 10.1371/journal.pone.0259036

**Published:** 2021-10-27

**Authors:** Diah Harnoni Apriyanti, Luuk J. Spreeuwers, Peter J. F. Lucas, Raymond N. J. Veldhuis

**Affiliations:** 1 Faculty of EEMCS, University of Twente, Enschede, The Netherlands; 2 Indonesian Institute of Sciences (LIPI), Jakarta, Indonesia; 3 LIACS, Leiden University, Leiden, The Netherlands; Wuhan University of Science and Technology, CHINA

## Abstract

The color of particular parts of a flower is often employed as one of the features to differentiate between flower types. Thus, color is also used in flower-image classification. Color labels, such as ‘green’, ‘red’, and ‘yellow’, are used by taxonomists and lay people alike to describe the color of plants. Flower image datasets usually only consist of images and do not contain flower descriptions. In this research, we have built a flower-image dataset, especially regarding orchid species, which consists of human-friendly textual descriptions of features of specific flowers, on the one hand, and digital photographs indicating how a flower looks like, on the other hand. Using this dataset, a new automated color detection model was developed. It is the first research of its kind using color labels and deep learning for color detection in flower recognition. As deep learning often excels in pattern recognition in digital images, we applied transfer learning with various amounts of unfreezing of layers with five different neural network architectures (VGG16, Inception, Resnet50, Xception, Nasnet) to determine which architecture and which scheme of transfer learning performs best. In addition, various color scheme scenarios were tested, including the use of primary and secondary color together, and, in addition, the effectiveness of dealing with multi-class classification using multi-class, combined binary, and, finally, ensemble classifiers were studied. The best overall performance was achieved by the ensemble classifier. The results show that the proposed method can detect the color of flower and labellum very well without having to perform image segmentation. The result of this study can act as a foundation for the development of an image-based plant recognition system that is able to offer an explanation of a provided classification.

## Introduction

Identifying a plant is not an easy task, not even for the expert. There are many features of plants that play a role in this task. Color is often used as one of the more important features in flower recognition using image processing [1]. This feature also appears in descriptions used in *identification keys*, i.e., structured features to identify a species [2]. In this paper we investigate the role color can play in identification, where digital photographs in conjunction with descriptions of orchids are used as the experimental domain. Although color is just one of the features deployed in orchid identification, it is not known how hard it is to detect the colors of an orchid in an image. This is the research question which this paper aims to answer. Our future plan is to build an explainable flower identification computer-based system in which color is one of the features used to support the explanation of why a particular species is most likely.

**Fig 1:**
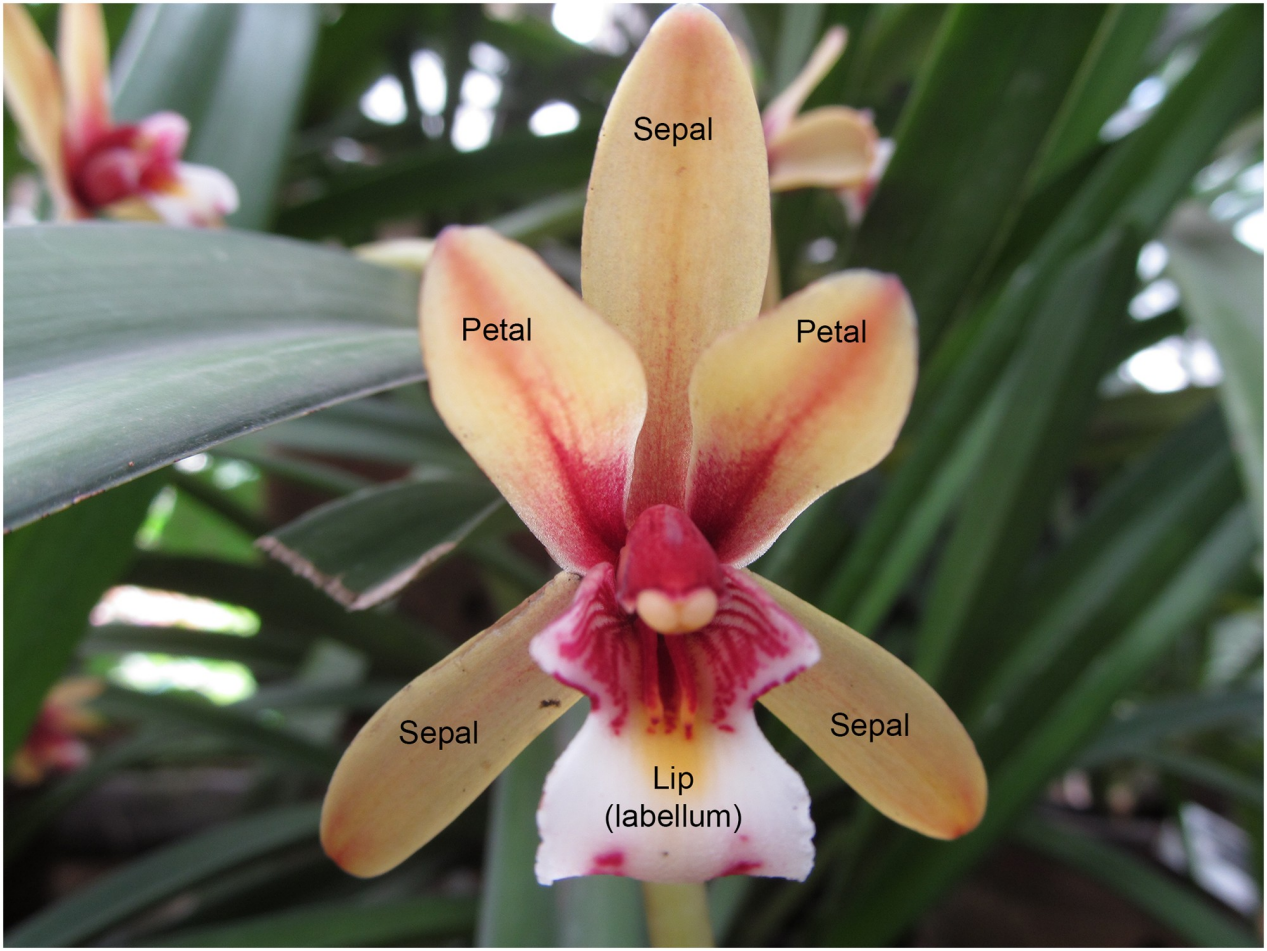
Parts of an orchid flower. The flower is made up of sepals and petals; there is only one labellum and it may have its own separate color.

The colors of some parts of an orchid—the sepals, the petals, and the lip as shown in Fig 1—are of particular value when identifying a plant. In this research, the sepals and petals together are called the **flower**, while the lip, that acts as a landing platform for insects, is called the **labellum**. It may be expected that the difficulty of color detection is not the same for the flower and labellum, because of differences in shape and size. Automatic color detection is not straightforward, as both flower and labellum may contain multiple colors, e.g. red and green, increasing the size of the search space, yet in the face of the availability of only a limited number of photographic images of orchids with varying image quality. In addition, the flowers pictured in the images are normally surrounded by other plants, trees, grass, etc., sometimes making even the detection of the flowers in the image a challenge. As the flowers are not photographed in a standardized fashion, it does not appear easy to develop a segmentation algorithm that is able to distinguish the flowers from the background. Of course, often the photographers did their best to obtain a good (most of the characteristic details of the flower can be distinguished) and clear image of the flowers. However, we noticed that not all photographs are of good quality and even the human eye sometimes has difficulty to find the flowers in the image.

The context of the present research is that of orchid databases (e.g., https://gobotany.nativeplanttrust.org/), nowadays widely available and online accessible through the internet. They contain systematic descriptions of orchids, identified by species name and classified in genera, in terms of specific features (color of the flowers and labellum, shape and texture of the leaves, geographical location where they are found, etc.), complemented by multiple digital photographic images of the plant. As existing image-based flower recognition systems offer black-box approaches, which only output the species name [3–5], we believe that the way a taxonomist identifies an orchid, i.e., in terms of its features, is a valuable source of inspiration for designing a plant species identification computer-based system.

In the present paper, a novel approach to automated color detection of flowers in images is proposed; all of the published studies in image-based flower recognition have used **color moments** and **color histograms** for extracting color features from images [6]. In contrast, in our research **color labels** are used, i.e., names of colors used to describe objects, in our case flowers. Taxonomist have used color labels, together with other flower features, to describe the characteristics of flowers for centuries [7]. Thus, in the present paper there are two novel contributions:

- Firstly, **we have built a new format** for a flower-image database, which not only consists of flower images, but in addition of descriptions of the flower characteristics.
- Secondly, we propose **a new approach to extract color features from flower images using color labels and deep learning without making use of image segmentation**.

To achieve a reliable color detection system, we conducted experiments with five different deep learning architectures, with transfer learning and varying amounts of unfreezing of neural network layers, and various classifier methods (multi-class classifiers, combined binary classifiers, and ensemble classifiers), using different color scheme scenarios. Our experiments show that the ensemble classifier outperforms other classifiers.

## Materials and methods

### Related work

In this section, we briefly review the most relevant researches on: (1) color detection [8–14]; and (2) flower classification using deep learning [3, 5, 15–20].

Color is widely used as one of the features in object recognition and various methods are employed in identifying color in images. Popular methods are **color moments** and the **color histogram**. **Color moments** measure the color similarity between images by using mean, standard deviation, and skewness [8], whereas the **color histogram** is based on the computation of the frequency with which colors occur in an image. To compute a color histogram, several color space options can be chosen from, such as the RGB color space, HSV color space, CIE L*a*b* color space, etc. [9].

Besides color moments and the color histogram, another currently popular method is based on **color labels**. There are two popular methods available to assign linguistic color labels to image pixel, using either chip-based color names or real-world image-based color names. **Chip-based color names** are obtained by mapping RGB values to the color names of a labeled set of color chips. It works well when the image is taken under conditions of ideal lighting. There are some chip-based color naming references that have been built from different dataset [10–12]. Alternatively, **real-world image color names** are used, where color names are learned from objects in real-world images. Van der Weijer et al. [13] have done research employing this method. They used Probabilistic Latent Semantic Analysis (PLSA), a generative model introduced by Hofmann [14] for document analysis, to obtain the distributions of the color names over L*a*b* values. They claim that their method is photometrically robust because the images for learning have been taken from the internet using Google Search with varying illuminants, cameras, and camera settings. However, in contrast to our approach, the method requires segmentation of an object in an image and the segmented object’s color is subsequently determined by counting the number of color pixels. Furthermore, the method is unable to differentiate between the color of the flower and labellum, which is one of the aims of our research.

These limitations brought us to proposing a new method to identify the color of flower and labellum by color labels and deep learning. Basically, we adopted the idea to start with real-world images as suggested by Van der Weijer [13]. Instead of deploying images from general objects (such as cars, shoes, dresses, etc.), we used only flower images. To decide on the color name, we employed deep learning instead of PLSA.

In recent years, flower classification by means of **deep learning** has been evolving rapidly. Hiary, et al. have proposed a two-step deep-learning method to classify flower species [5]. The first step consists of segmenting the flower region using a Fully Convolutional Network (FCN), composed of 5 blocks from the VGG16 architecture [21] and an additional three de-convolutional layers. The second step is concerned with classifying the type of flower using a Convolutional Neural Network (CNN), which also uses the VGG16 architecture, followed by 3 convolutional layers with 512 feature maps. They chose VGG16 because it better suits the flower classification task compared to other deep-learning methods. Evaluation of the performance of their method was conducted on three different datasets, two from Oxford—the Oxford 17 and the Oxford 102 dataset —, and the Zou-Nagy dataset. The results show that their proposed method can achieve at least an accuracy of 97% on these datasets.

Gurnani et al. have compared the performance of the GoogleNet and AlexNet architecture in classifying different flowers from the Oxford 102 dataset [15]. The GoogleNet architecture uses Inception as the backbone, while AlexNet uses eight layers with the first 5 layers being convolutional layers and the last 3 layers being fully connected layers. They kept the same hyper-parameters during training of both architectures, concluding that the GoogleNet yielded a better performance than the AlexNet.

Use of the Inception-v3 feature extractor with transfer learning, together with a CNN, was proposed recently by Arwatchananukul et al. in an attempt to distinguish 15 species of Paphiopedilum orchids [16]. They also built a new Paphiopedilum orchid database consisting of 1500 images, 100 images per species, each of them front-view images with the flower and labellum placed in a very similar standardized way. The performance of their classification system reached as highest accuracy a value of 98.6%. Inception-v3 was also used by Xia et al. [17] for flower classification. They used Oxford-17 and Oxford-102 flower dataset in their experiment. The results showed that the system can greatly improve the accuracy of flower classification.

In Liu et al. [3], two network architectures, VGG16 and ResNet50, were applied to recognize Chrysanthemum flowers. They used two datasets for training and evaluation. The dataset for training consisted of 14,000 images with 103 cultivars, while another dataset, comprising 197 images, was deployed for evaluation. Deep learning was selected as method in this research because of its potential advantage of achieving a good performance.

Furthermore, a research in Orchid flowers recognition has been conducted by Zhang et al. [18]. They built a dataset comprising two million orchid images from 2608 species and proposed a joint framework between deep learning and a tree classifier to identfy plant species in large scale. AlexNet was used for deep learning architecture, while two-layers tree classifier was constructed which can perfectly organize the large number of plant species. The results showed that the proposed framework can achieve very competitive results in accuracy and computational time.

Research to compare the performance of CNNs combined with transfer learning against other machine learning methods using handcrafted feature extraction was initiated by Gogul and Kumar [19]. They used the Inception-v3, Xception, and Overfeat architecture to do feature extraction. In deep learning, specifying the features one by one as normally done in handcrafting is not needed. The handcrafted features that are often used in flower recognition are: color, shape, and texture. All of these features acted as input to the machine learning method. Decision trees, k-nearest neighbor, naive Bayesian networks, and random forests were compared to each other. The results showed that in extracting features, deep learning outperformed the other machine learning methods (using handcrafted feature extraction) in terms of accuracy. In this case, the highest accuracy was achieved by the Inception-v3 architecture.

A comparison of the performance of some deep learning architectures is also made in the work by Basa et al. [20]. In this research, they compared the performance of VGG16, ResNet-50, MobileNet, DenseNet, and NasNet-Mobile combined with a fine-tuning method deploying some datasets including the Oxford-102 Flower dataset. The results showed that VGG16 and ResNet-50 achieved the highest performance, in contrast to NasNet that performed poorly. However, in several studies [22, 23], NasNet, which is a relative new architecture in deep learning, often gives the highest performance.

Although the summary of related research above certainly conveys the impression that much progress has been made during the last decade by applying deep learning to flower classification, in our research we explicitly aim to move away from the black-box nature of deep learning, by providing information about the role of each of the features exploited in the classification process, although these features may be identified by deep learning. In this paper, the feature studied is color.

### The color of orchids

#### Relevant features

Different from the situation with other flowering plants (angiosperms), the color of the labellum is employed as extra information by taxonomists, in addition to the color of the flower, for describing the characteristics of the orchid. Thus,

- **Color of Flower**, CF for short, and
- **Color of Labellum**, abbreviated to CL,

are the color features of orchids that have been selected for our research because of their easy identifiability by both humans and computer vision systems. In the following, these color features will be abbreviated *together* as ‘CO’. Both CO features have an associated *domain* (range of values), denoted *D*(CO). For the domain of these CO features, we have designed two scenarios. The first scenario is using non-binary, *multinomial* data. For example, the variable CF (Color of Flower) can take color values such as ‘red’, ‘purple’, ‘yellow’, etc. The other, simpler *binary* scenario assumes that one of the colors is taken as the indicator, e.g., ‘red’, whereas the other value is ‘non-red’ (in general, ‘color’ and ‘non-color’). Below, first the design of multinomial color schemes will be described, which will be followed later by the design of multi-class and binary classifiers, where the latter deal with the binary scenario.

#### RGB encoding

**Fig 2:**
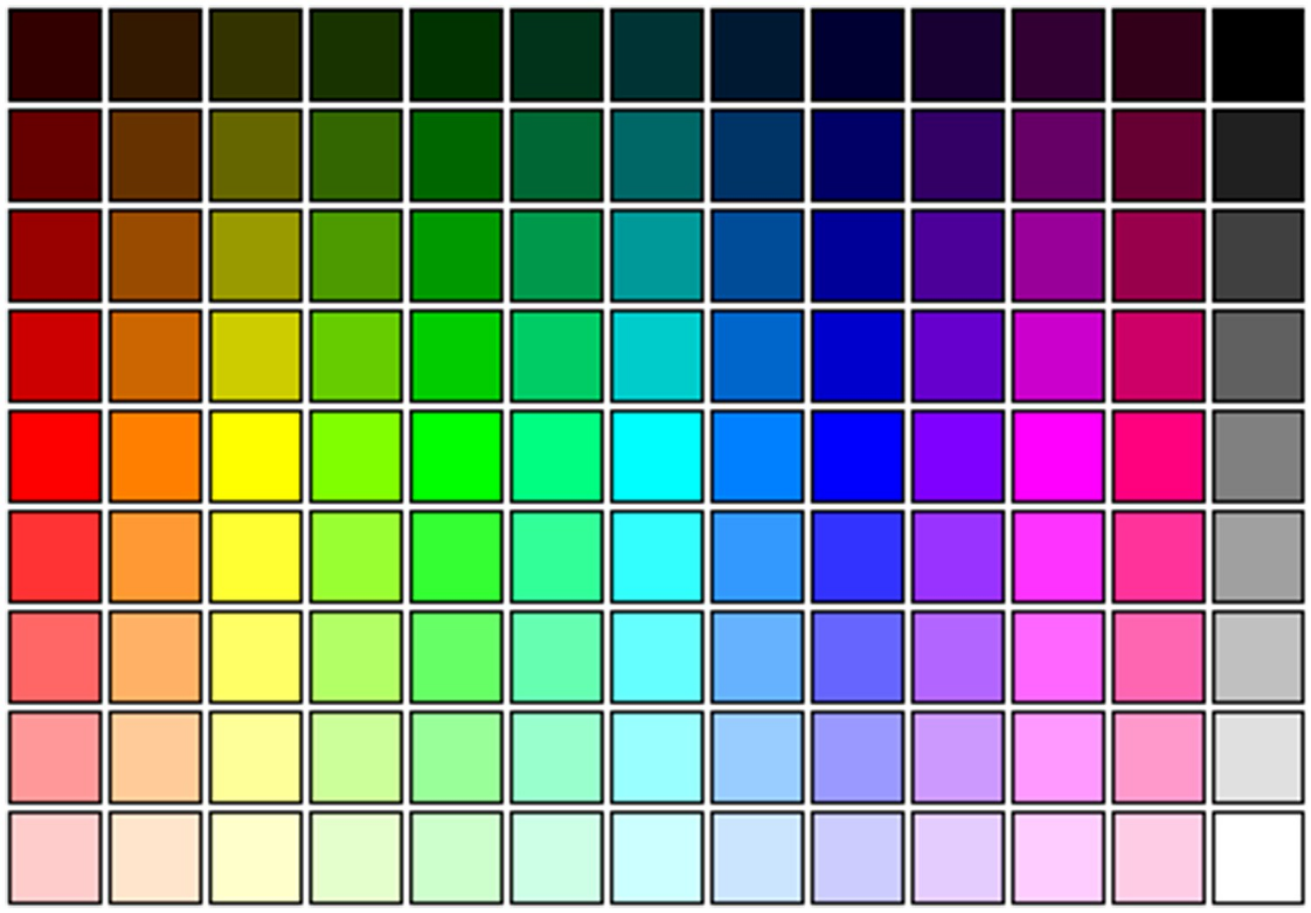
RGB color model as 9 × 13 matrix *M*. White is located at the right bottom cell; the blue color, columns 7–9, does not occur in our orchids.

We use the RGB (Red Green Blue) color space as a foundation for the color labeling as this color model is also used in the description of the digital images of orchids. RGB color labeling was applied to the color labeling of CF and CL. As the RGB color model describes a 3-dimensional space, we used the 2-dimension matrix mapping, shown in Fig 2, to facilitate designing a mapping.

**Fig 3:**
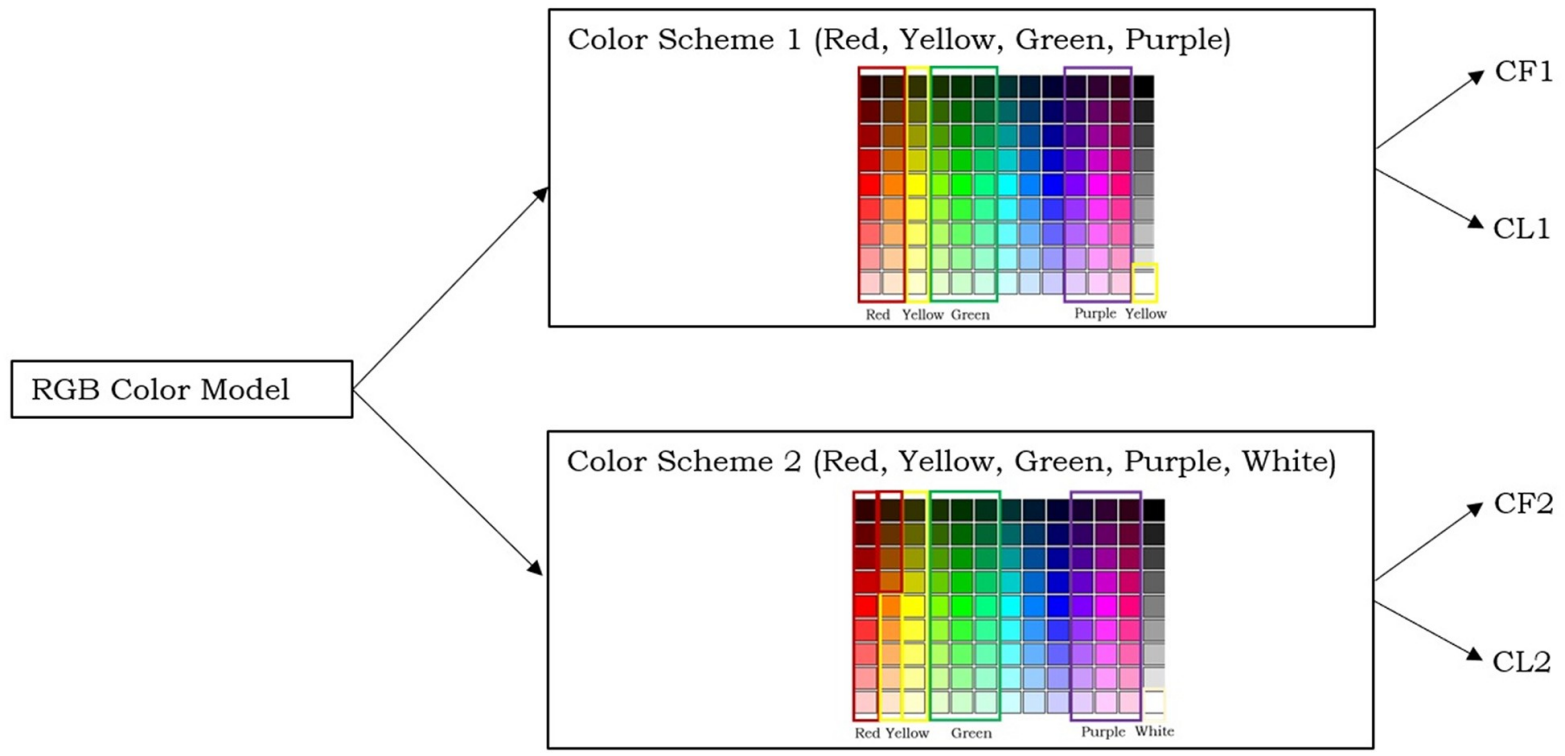
Color scheme scenario.

To reduce the search space, we designed two color schemes from the RGB color model as described in Fig 3.

One alternative (called below **color scheme 1**) investigated is based on the following colors:

- **Red** (standing for redish, i.e., red, brown, and orange): first 2 columns of the matrix of Fig 2.
- **Yellow** (standing for yellow, light yellow, and white): 3rd column of the matrix of Fig 2.
- **Green** (standing for greenish): columns 4–6 of the matrix of Fig 2.
- **Purple** (standing for purple to pink): columns 10–12 of the matrix of Fig 2.

As blue neither occurs in a flower nor in a labellum of an orchid, columns 7–9 are ignored. Finally, note that white is actually the rectangle *M*[9, 13], but *M*[9, 3] (the end of the Yellow column) is close to white. This choice results in 4 different colors.

The other alternative (**color scheme 2**) investigated was to employ the following colors:

- **Red** (standing for redish, i.e., red and brown): first column of the matrix of Fig 2 and *M*[1 : 4, 2].
- **Yellow** (standing for orange, yellow, light yellow): 2nd and 3rd column of the matrix of Fig 2, excluding *M*[1 : 4, 2], which is classified as red.
- **Green** (standing for greenish): columns 4–6 of the matrix of Fig 2.
- **Purple** (standing for purple to pink): columns 10–12 of the matrix of Fig 2.
- **White**: element *M*[9, 13] in the color matrix.

As again blue neither occurs in an orchid flower nor in its labellum, columns 7–9 are ignored. This choice yields 5 different colors, one color more than color scheme 1. Also observe that only two of the color names of color scheme 1 and 2 have exactly the same semantics: green and purple; the meaning of the other color names is different, which is worth to remember as otherwise it may make the rest of the paper confusing. In the following, the color schemes in relationship to CF (Color of Flower) and CL (Color of Labellum) are referred to be CF1 and CF2, respectively, and CL1 and CL2, respectively.

#### Primary and secondary color combinations

Often flowers and labellums have multiple colors; the color combination may help in identifying them. One option is to use a multi-label classification method (with the possibility of having two or more color labels at the same time). However, this would imply that we had to combine two (or more) colors according to the Cartesian product of their domain, *D*(CO) × *D*(CO) for two colors, yielding 16, e.g. (Red, Yellow) and (Yellow, Red), and 25 colors, respectively, based on color scheme 1 and 2. As we only had a dataset of limited size, we decided to investigate whether the number of labels could be reduced.

As the color of a flower is often not unique, we have defined the variable CO as a subset of the Cartesian product of two other color variables called CO_*p*_ and CO_*s*_, respectively, i.e., *D*(CO) ⊆ *D*(CO_*p*_) × *D*(CO_*s*_), with CO_*p*_ the primary color and CO_*s*_ the secondary color. Based on the description of orchids, the primary color CO_*p*_ has a domain with eight values: blue, brown, green, pink, purple, red, white, and yellow; the secondary color CO_*s*_ has a domain consisting of seven values: brown, green, pink, purple, red, white, yellow. The advantage of this definition of CO is that unlikely or impossible color combinations can be left out of the definition, and do not have to be assessed during statistical estimation of the probability distribution.

The order of colors is significant as the first, primary color name, will fill most of the flower or labellum area, and the secondary color a smaller part, often only a rim. However, to reduce the number of colors we assume **commutativity** of their combination, i.e., for colors *A*, *B* we assume that *AB* = *BA*. This choice implies that we can compute the number of color combinations with repetitions (the primary and secondary colors can be the same, which is the same as that there is no secondary color), *k* at the time, of *n* colors:

$$\left(\begin{array}{c} n+k-1\\ k \end{array}\right)$$ (1)

As in this case we wish to model image colors, not the colors in the descriptions (although there is a strict mapping between the two), we assume that we have to handle 4 and 5 colors, respectively, using the color schemes previously mentioned. Thus, in the present situation for **color scheme 1** *n* = 4 and *k* = 2, hence: $\left(\begin{array}{c} 5\\ 2 \end{array}\right)=10$. The values include, for example, RedRed, which is just Red (the entire flower is red, there is no secondary color). Finally, some of the combinations do not occur in nature. This yields the following combinations:

- (1) RedRed = Red
- (2) YellowYellow = Yellow
- (3) GreenGreen = Green
- (4) PurplePurple = Purple
- (5) RedYellow = YellowRed
- (6) RedGreen = GreenRed
- (7) RedPurple = PurpleRed **X**, **Y**
- (8) YellowGreen = GreenYellow
- (9) YellowPurple = PurpleYellow
- (10) GreenPurple = PurpleGreen **X**, **Y**

Two combinations, indicated by **X**, do not occur in practice for the variable CL (Color of Labellum), and **Y** for CF (Color of Flower), hence what remains are **8 combinations** of primary and secondary colors.

For **color scheme 2** we have *n* = 5 and *k* = 2, thus $\left(\begin{array}{c} 6\\ 2 \end{array}\right)=15$.

- (1) RedRed = Red **X**
- (2) YellowYellow = Yellow
- (3) GreenGreen = Green
- (4) PurplePurple = Purple
- (5) WhiteWhite = White
- (6) RedYellow = YellowRed
- (7) RedGreen = GreenRed
- (8) RedPurple = PurpleRed **X**, **Y**
- (9) RedWhite = WhiteRed **X**, **Y**
- (10) YellowGreen = GreenYellow **X**
- (11) YellowPurple = PurpleYellow
- (12) YellowWhite = WhiteYellow **Y**
- (13) GreenPurple = PurpleGreen **X**, **Y**
- (14) GreenWhite = WhiteGreen
- (15) PurpleWhite = WhitePurple

**Fig 4:**
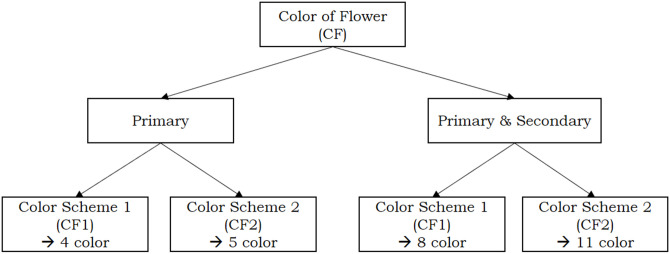
Scenarios for Color of Flower (CF).

**Fig 5:**
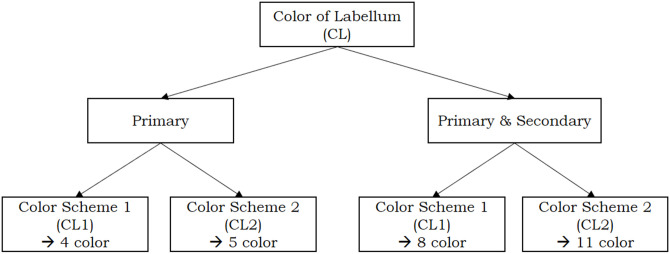
Scenarios for Color of Labellum (CL).

Five combinations, indicated by **X**, do not occur in practice for the variable CL (Color of Labellum), hence what remains are **10 combinations** of primary and secondary colors. For the variable CF (Color of Flower) we can remove the 4 combinations indicated by **Y**, yielding **11 combinations**. To make it easier for the reader to distinguish the employed color scenarios, the diagrams depicted in Figs 4 and 5 are included in the paper.

### Orchid dataset

A dataset was composed by us that includes 7156 images of orchid flowers, consisting of 156 different orchid species. Most of the images were Flickr images under the Creative Commons license, downloaded by us through the Flickr API. Some of the images were obtained from websites such as Go Botany (Native Plant Trust) and the Encyclopedia of Life (EoL). Different from other flower image datasets, in addition to the images our dataset contains, descriptions are included of the features for each orchid. The feature descriptions were obtained from the “Go Botany” and “Go Orchids” websites. Several features are used in this dataset: colors, texture, inflorescence, number of flower, and labellum characteristics. However, in this paper, we only focus on the color features.

**Fig 6:**
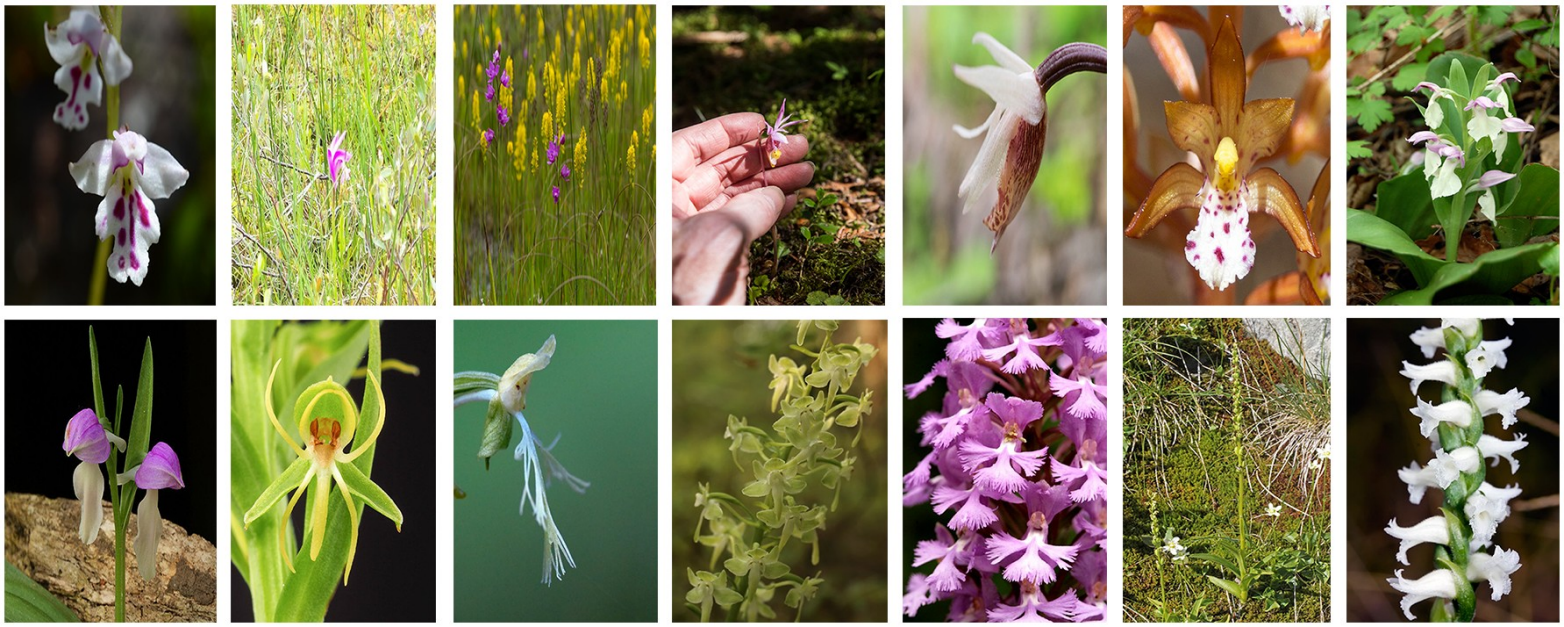
A selection of pictures of orchids in the dataset used in our research.

The dataset is quite challenging because it suffers from imbalance: some classes are covered by a large number of images, whereas other image classes are underrepresented, i.e., only appear in very small numbers. However, this imbalance in the data might be caused by the rareness of the species in nature, leading to a lack of pictures of certain species. Besides that, the dataset contains flower photographic images with a natural background, taken from various position and angles, with varying conditions of illumination and noise, rendering this dataset non-uniform and thus hard to analyze (see Fig 6 for some example pictures).

The dataset used in this paper can be downloaded at https://doi.org/10.7910/DVN/0HNECY [24].

### Deep learning

As deep learning appears to be one of the best available methods for image interpretation, we compared the performance of some promising deep learning architectures on our orchid dataset with the hope of discovering the best architecture for our color detection system. We explored VGG16 [21], Inception-v3 [25], Resnet50 [26], Xception [27], and NasNetLarge [22]. Rather than training a custom-made convolutional network from scratch, transfer learning using these pre-trained architectures, trained on a large dataset (in our case the ImageNet dataset), were used. A pre-trained network is in particular attractive if only a small dataset is available.

In transfer learning, we can freeze or unfreeze the layers in pre-trained model to obtain the best performance. Table 1 shows the number of layers in each architecture. Based on the table, some experiments were performed by adjusting the number of frozen layers in the pre-trained models. We froze $\frac{3}{4}$th, $\frac{1}{2}$, and $\frac{1}{4}$th of the bottom layers. In addition, we also tried to freeze the first layer and unfreeze all of the layers.

**Table 1:** The depth of the network model.

Network model	Number of layers
VGG16	19
Inception-v3	311
Resnet50	175
Xception	132
NasNet	1039

We added three new layers in the last layer of pre-trained model. The last new layers were: a flatten layer, a dense layer with 512 neurons, a dropout layer with a probability of 0.5, and as last dense layer one with **the number of neurons equal to the number of colors that we wished to detect**. ReLU was employed as the activation function in the first dense layer and softmax as the activation function for the last dense layer.

The hyper-parameter for deep learning that we employed in the experiments are shown in Table 2. As input to the neural networks acted an RGB image with size 224 × 224 for all of the architectures except NasNetLarge which uses 331 × 331. We used a batch size equal to 64 and the number of epochs was equal to 100. To achieve a better performance we also fine-tuned our pre-trained models using data augmentation. *Data augmentation* is a method to increase the diversity of the training set by applying random transformations. The transformations we applied to the dataset were: rotation, shrink, flip, and zoom. The *class weight method* was applied to the data to obtain more balanced data; it works by replicating the smaller (in number of instances) class until as many samples are obtained as for the larger class. Adam was the optimizer used in this case, with as loss function: weighted binary cross entropy. Software was developed for the experiments based on the software libraries tensorflow and keras, on top of the scripting language python [28, 29].

**Table 2:** Setting of deep learning architecture.

Hyper-parameters	Value
Optimization algorithm	Adam optimizer
Initial LR	0.00005
Epochs	100
Batch size	64
Image Input Size	224 × 224 331 × 331 (NasNet)
Loss function	Weighted binary cross entropy

### Color classifier methods

As is clear from the description above, the feature of color of both flowers and labellum are described by more than two class labels. Hence, the classifier models we need to learn from the data are of the form $C:R^{p\times q}\to \left\{1,\ldots ,m\right\}$, p,q,m∈N, where an image $I\in R^{p\times q}$ is described by a *pq*-dimensional (here 224 × 224; 331 × 331 for NasNetLarge) real matrix, and for primary colors only, *m* = 4 using color scheme 1, and *m* = 5 for scheme 2; when we consider combining primary and secondary colors, *m* = 8 for scheme 1 and *m* = 10 or *m* = 11 for scheme 2, as discussed above.

**Fig 7:**
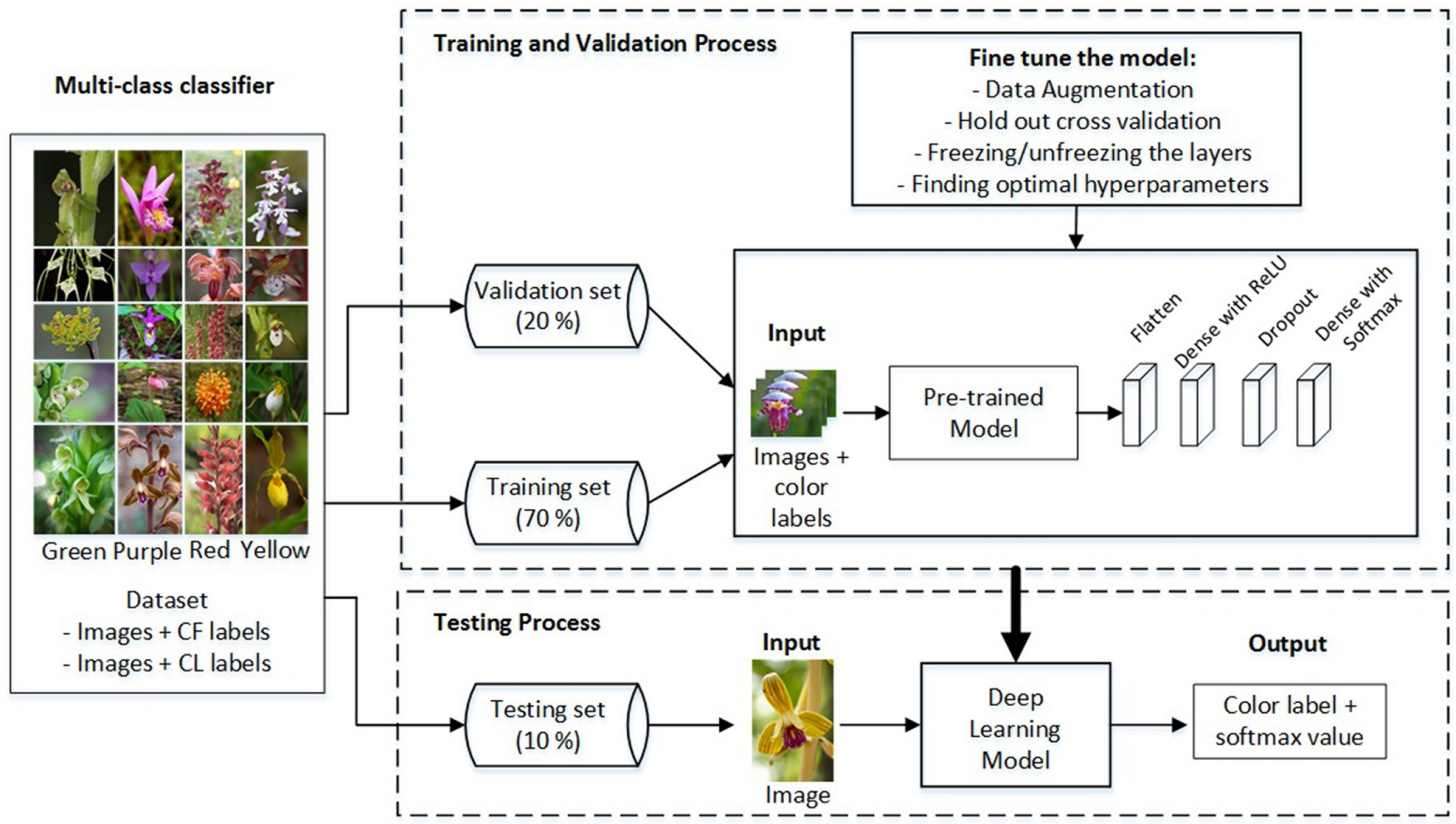
Multi-class classifier.

There are multiple ways in which color classification can be handled. The first way is that one simply learns a color **multi-class classifier** *C*. Fig 7 shows the framework for multi-class classifier using deep learning. After dividing the dataset into some training set, validation set and testing set, we use the flower images together with its color labels as the input and train them using multi-class classifier based on one of deep learning architecture. In multi-class classifier, we only need one classifier for predicting various color labels. The outputs for both training and testing are color label and softmax value. The disadvantage is that in situations of a small dataset, it is hard to learn *C* when the number of color labels is large, such as 10 and 11 in our case. An alternative solution is to learn multiple **binary classifiers**, $C_{i}:R^{p\times q}\to \left\{0,1\right\}$, *i* = 1, …, *m* − 1 and to combine them.

Let $I\in R^{p\times q}$ be an *image* and let us denote by *C*_*i*_ the situation that *C*_*i*_(*I*) = 1 and by ¬*C*_*i*_ the case that *C*_*i*_(*I*) = 0. Note that the situation where we know nothing about the actual color of a flower or labellum can be summarized by the following logical disjunction (with ∨ having the meaning of inclusive OR):

$$\begin{array}{c} \Gamma \equiv (C_{1}\vee C_{2}\vee \cdots \vee C_{i}\vee \cdots \vee C_{m}) \end{array}$$ (2)

called the *domain closure axiom* in artificial intelligence [30], which is augmented with *mutual exclusiveness*, ¬*C*_*i*_∨¬*C*_*j*_, 1 ≤ *i*, *j* ≤ *m*, *i* ≠ *j*. It simply means that known is that the actual color is one of the allowed colors, and having two or more colors at the same time is inconsistent. Note the difference with totally knowing nothing; we do know something, but not yet which specific color the plant part has. This formula plays an essential role below.

We have the following potential results from the merge of the binary classifiers:

- (¬*C*_1_∧⋯∧¬*C*_*i*−1_∧*C*_*i*_∧¬*C*_*i*+1_∧⋯∧¬*C*_*m*−1_∧¬*C*_*m*_), where *C*_*i*_ is the *only* established positive label for image *I*, ¬*C*_*k*_, *k* = 1, …, *m* − 1, *k* ≠ *i*, is the output of classifier *k*, and ¬*C*_*m*_ is obtained by the mutual exclusiveness axioms;
- (¬*C*_1_∧⋯∧*C*_*i*_∧⋯∧*C*_*j*_∧⋯∧¬*C*_*m*−1_), with *i* ≠ *j*, meaning that the binary classifiers yield contradictory (more than one positive label) result. The label is unknown in that case.
- (¬*C*_1_∧⋯∧¬*C*_*i*_∧⋯∧¬*C*_*m*−1_), which when combined with Γ yields that *C*_*m*_ is the right label of the image.

This way of combining the result of multiple binary classifiers is known as the *one-versus-the-rest* classifier [31].

A third alternative is to learn an approximate probability function $f_{i}:R^{p\times q}\to \left[0,1\right]$ for each color *i* = 1, …, *m* by deep learning and to select the color *k* where an image *I* has **maximum probability**:

$$\begin{array}{c} k={\text{argmax}}_{1\leq i\leq m}f_{i}\left(I\right) \end{array}$$ (3)

The fourth and last classifier we wish to consider is that of combining a multi-class and combined-binary classifier as an **ensemble** [31]. Often ensembles of classifiers use some kind of voting mechanism to determine the output. As in this case the ensemble consists just of two classifiers, we have designed two ways to determine which class label to yield as a result. Let *C*_1_ and *C*_2_ be the two classifiers that make up the ensemble. The following heuristics have been designed (and will be evaluated below) yielding two different ensemble methods:

- **Most likely true color** (MLTC) ensemble. If both *C*_1_ and *C*_2_ produce the same label as output, then this is taken as the result. However, when the labels are different, we select the color of *C*_1_ or *C*_2_ that has the highest true positive rate (TPR; see next section for its definition).
- **Most likely color ratio** (MLCR) ensemble. Similar to the MLTC ensemble, if both *C*_1_ and *C*_2_ produce the same label as output, this is taken as the result. However, when the results are different, we decide to produce the color for which the true positive rate ratio of these two colors between the two classifiers is highest as output. The ratio is interpreted in this case as a heuristic saying that if the difference in ratio between a specific color of a classifier is larger than for the other classifier, then the best color choice is the one with the lowest ratio.

An example may be valuable to help in understanding of what we have just described.

**Example 1** *Consider the two classifiers C*_1_ *and C*_2_, *respectively, with outputs* red *and* white, *respectively, and the following results for the two different ensemble methods. For C*_1_ = red, TPR = 0.38, *whereas for C*_1_ = white, TPR = 0.70; *similarly, for C*_2_ = red, TPR = 0.58, *whereas for C*_2_ = white, TPR = 0.59. *If we use the MLTC ensemble method, the choice would be* white (*that is*, *C*_2_ *wins*) *as* 0.59 > 0.38. *The MLCR ensemble method will choose* red, *as the ratio for* white *is in this case equal to* 0.59/0.70 ≈ 0.84 *and for* red *equal to* 0.38/0.58 ≈ 0.66 *indicating that for red the values are wider apart than for white. Hence*, red *is the proper choice for the MLRC method*.

The detailed schemas to understand easily the scenarios for combined-binary classifiers and ensemble classifier can be seen in the S1 Fig.

### Training and testing procedures

We divided the dataset described above into two parts: one for training the classifiers, using a training and validation set, and the other one for testing purposes. The training and validation sets are used for optimization purposes, i.e., to fine-tune a model. The independent testing set ensures that testing is done on images that have not been met during training. The percentages of each part were approximately: 70% (training set), 20% (validation set), and 10% (testing set). We had 5119 images for training, 1235 images for validation, and 802 images for testing. Data augmentation was only applied in the training process. In this dataset, we used two color characteristics, CF and CL, as described in detail above. We extracted the primary color and also primary and secondary color from the images, using the color schemes described above, to handle the large number of flower species. Note that in training the images and associated color labels are used as input, in a form of supervised learning, whereas in testing only the images act as input and the associated color labels are only used to determine the classification performance of the various classifiers.

There are several measures in use to evaluate the performance of a classifier. As we are dealing with multi-class classification problems, no use is made of ROC analysis, which was originally designed for binary classification. Instead we will examine the performance by showing confusion matrices; they have the advantage that they include all the needed information to compute various performance measures, offering detailed insight into how well a classifier performs. A confusion matrix is also useful to visually show imbalance in a dataset.

A **confusion matrix** is computed by summarizing the number of correct and incorrect predictions per class. There are two kinds of confusion matrices. The first kind is a confusion matrix with entries computed by directly placing the number of correctly or incorrectly predicted cases into the table. Although we will use confusion matrices for multi-class classification, which do not have the 2 × 2 table structure as for binary classification, we illustrate the basic ideas by this simplest possible confusion matrix. TP stands for ‘True Positive’, representing the number of cases with positive classes that were predicted correctly as being positive. TN stands for ‘True Negative’; it represents the number of cases with negative class that is predicted correctly as being negative. FP is short for ‘False Positive’, being the number of cases with negative class that the classifier predicted as being positive. Finally, FN stands for ‘False Negative’, being the number of cases with positive class that are predicted as being negative. Table 3(a) summarizes these measures in one matrix.

**Table 3:** The confusion matrices; (a) without normalization and (b) normalized.

	Predicted Value			Predicted Value	
**Actual Value**	TP	FN	**Actual Value**	TP/TP + FN	FN/TP + FN
	FP	TN		FP/FP + TN	TN/FP + TN
(a)			(b)		

The second kind of confusion matrix includes *rates* or frequencies, based on the data in the *unnormalized* confusion matrix in Table 3(a) which is computed by dividing the number of correctly or incorrectly predicted cases by the total number of cases per class. If we need to obtain a *normalized* confusion matrix, we only need to divide each entry of the confusion matrix by the total number of cases per class like in as shown in Table 3(b). For example, the number of true positives (TP) is turned into the TPR (True Positive Rate) by its definition in the upper-left table entry.

Another often used measure is ‘**accuracy**’: the total number of correct predictions divided by the total number of cases. The accuracy can be calculated directly from the confusion matrix as follows [32]:

$$\begin{array}{c} \text{accuracy}=\frac{\text{TP}+\text{TN}}{\text{TP}+\text{FP}+\text{TN}+\text{FN}} \end{array}$$ (4)

In addition, the performance measure ***F***_1_ is applied frequently, which defined as the harmonic mean of Recall and Precision [32]:

$$\begin{array}{c} F_{1}=\frac{2\cdot \text{Recall}\cdot \text{Precision}}{\text{Recall}+\text{Precision}} \end{array}$$ (5)

where

$$\begin{array}{c} \text{Recall}=\frac{\text{TP}}{\text{TP}+\text{FN}} \end{array}$$ (6)

and

$$\begin{array}{c} \text{Precision}=\frac{\text{TP}}{\text{TP}+\text{FP}}. \end{array}$$ (7)

In practice we will use the **macro-*F***_**1**_ measure, as it yields insight into the classification performance for the entire class variable, as it is defined as the mean of the $F_{1}^{i}$ measure for the individual classes *i*:

$$\begin{array}{c} \text{macro}-F_{1}=\frac{1}{n}\sum_{i=1}^{n}F_{1}^{i} \end{array}$$ (8)

where *n* represents the number of classes. Thus, henceforth, when we refer to *F*_1_, we actually mean macro-*F*_1_.

## Results

As mentioned above, we conducted the experiments using different deep learning architectures to find the pre-trained model that performed best on our orchid data. Using the best pre-trained model, we then conducted further experiments by using two color schemes for primary color only and on relevant combinations of primary and secondary color, where the color schemes are referred to in both cases as CF1, CF2, for color of flowers, and CL1 and CL2, for color of labellum. Three different types of classifier were trained and tested using the data: multi-class classifiers, combined binary classifiers, and ensemble classifiers.

### Selection of a pre-trained deep-learning model

**Fig 8:**
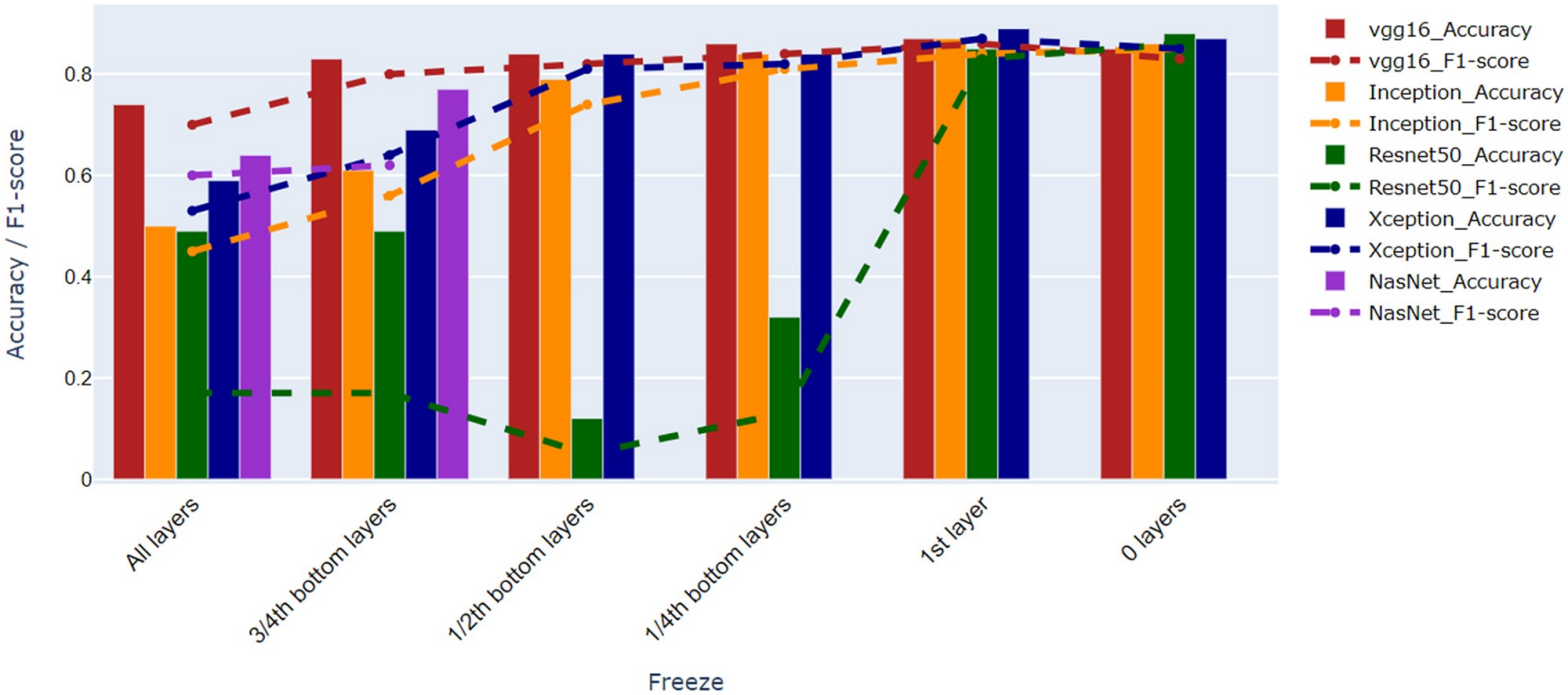
The performance of deep learning architecture on orchid flower dataset.

As detection of the color of the labellum is a more difficult problem than that of the flower, because of its smaller size, the choice of the architecture was guided by their capability of dealing with this color detection problem. Fig 8 shows the results for different architectures applied to our orchid dataset using the primary color of the labellum, using the CL1 color scheme. When all layers are frozen, each of the pre-trained models offers no more than 70% accuracy, except VGG16, which already is a good feature extractor. Its performance is relatively stable for all freezing and unfreezing scenarios. The performance of the other pre-trained models did not give significant improvement when we tried to freeze $\frac{3}{4}$th of the bottom layers. Inception-v3 and Xception give us a good performance since we freeze only $\frac{1}{2}$ of the bottom layers. In contrast, the performance of ResNet50 is decreasing when we freeze $\frac{1}{2}$ of the bottom layers. Their performance is quite significantly improving when freezing only the first layer and unfreezing the others, and remains good when unfreezing all layers. In the last two cases, all of the architectures give more or less similar performance. However, Xception gives us the best performance when we only freeze the first layer. Because of that, **from now on we will use Xception to conduct further experiments using different color schemes**.

### Computation times

For training the deep-learning models, a high performance computing system with a graphic processing unit was employed, giving rise to greatly reduced computation times. Training time was around three hours, while the testing process was very fast. There are were no significant differences in training time between VGG16, Inception, Xception, and Resnet50. For NasNet, we ran out of memory when trying to unfreeze the layers.

The results for the different classifiers are discussed next.

### Results for the multi-class classifiers

The results of the various multi-class classifiers in terms of accuracy and the *F*_1_-score are shown in Table 4. From the table, we conclude that color scheme 1 yields better accuracy and *F*_1_-score for color of labellum (CL), whereas color scheme 2 works better for color of flower (CF). As the results obtained for the detection of primary and secondary color together are worse than that for primary color only, the detection of the combination of colors is clearly more difficult than detecting one color only, which is according to expectations. Certainly part of the decrease in performance is due to the fact that the number of class labels is much higher for the color combination than for primary color only.

**Table 4:** Accuracy and *F*_1_ for the multi-class classifiers.

Color Combination	Color Scheme	Accuracy	F_1_
**Primary**	**CF1**	0.872	0.873
	**CF2**	**0.873**	**0.873**
	**CL1**	**0.888**	**0.874**
	**CL2**	0.855	0.823
**Primary and Secondary**	**CF1**	0.848	0.813
	**CF2**	**0.852**	**0.818**
	**CL1**	**0.848**	**0.773**
	**CL2**	0.817	0.751

Bold font is used to indicate clearly superior performance.

Based on these results, we decided to proceed developing separate binary classifiers for individual colors (a kind of ‘color specialists’), which would be subsequently be combined into multi-class classifiers, as described above in the section on color classifier methods.

### Results for the combined binary classifiers

Recall that for combining binary classifiers, we use two methods: one-versus-the-rest (**method 1**) and maximum probability (**method 2**). Table 5 offers a summary of the accuracy and *F*_1_-score for all combinations of color schemes and methods. Overall, both for primary, and primary and secondary color, method 1 yields lower performance in comparison to method 2. As Table 6 shows, the lower performance is often due to the unclassifier (inconsistent) cases, which is what we expected. The advantage of method 2 is that it always produces consistent classifications and thus below we will focus on this method.

**Table 5:** Accuracy and *F*_1_ for the combined binary classifiers.

Color Combination	Color Scheme	Method 1		Method 2	
		Accuracy	F_1_	Accuracy	F_1_
**Primary**	**CF1**	0.840	0.680	0.872	0.873
	**CF2**	0.819	0.679	**0.879**	**0.872**
	**CL1**	0.859	0.668	**0.887**	**0.857**
	**CL2**	0.822	0.674	0.864	0.837
**Primary and Secondary**	**CF1**	0.799	0.703	**0.863**	**0.833**
	**CF2**	0.763	0.659	0.865	0.810
	**CL1**	0.822	0.633	**0.854**	**0.735**
	**CL2**	0.724	0.578	0.814	0.681

Bold font is used to indicate clearly superior performance. Sometimes either accuracies or *F*_1_-scores were very close; then best performance choice was based on the best other measure.

**Table 6:** Accuracy of method 1 by including and excluding inconsistent results.

Color Combination	Color Scheme	Method 1	
		Accuracy (Include Inconsistent Results)	Accuracy (Without Inconsistent Results)
**Primary**	**CF1**	0.840	0.856
	**CF2**	0.819	0.847
	**CL1**	0.859	0.864
	**CL2**	0.822	0.854
**Primary and Secondary**	**CF1**	0.799	0.817
	**CF2**	0.763	0.817
	**CL1**	0.822	0.839
	**CL2**	0.724	0.793

Both CF and CL using primary colors show better accuracy than using primary and secondary color. However, the color schemes yield different results for CF and CL. When used to classify CL, we see that color scheme 1 appears to work better than color scheme 2. The opposite pattern occurs for CF for primary color, but not always, as shown for the color combination. Hence, it is clear that classifying color for flowers and the labellum are not task with identical difficulty, maybe because the labellum is much smaller than a flower; therefore, the simpler color scheme appears to work well for the labellum.

### Results for the ensemble classifiers

Because the results obtained by the combined binary classifiers were not *always* consistently better than those obtained by the multi-class classifiers, and the opposite was also not true, we decided to investigate two different, although related, ensemble classifiers, as described at the end of the section on the color classifier methods. Hence, two, closely related methods, the MLTC and MLCR methods, were compared to each other.

As can be noted from Table 7, in general, identifying color using MLCR yields better accuracy than MLTC. However, in some colors such as CL1 using primary color and using primary and secondary color, MLTC has the same performance as the MLCR method. Even, it slightly outperforms the MLCR method on CF2 using primary and secondary color. It appears that MLCR often has some positive, but slight, effect on the performance in comparison to MLTC, and sometimes not at all.

**Table 7:** Accuracy of MLTC and MLCR method on each color scheme.

Color Combination	Color Scheme	Accuracy	
		MLTC	MLCR
**Primary**	**CF1**	0.8691	**0.8753**
	**CF2**	0.8753	**0.8815**
	**CL1**	**0.8890**	**0.8890**
	**CL2**	0.8628	**0.8653**
**Primary and Secondary**	**CF1**	0.8566	**0.8616**
	**CF2**	**0.8691**	0.8678
	**CL1**	**0.8603**	**0.8603**
	**CL2**	0.8317	**0.8379**

Bold font is used to indicate clearly superior performance.

### Which classifier performed best?

**Fig 9:**
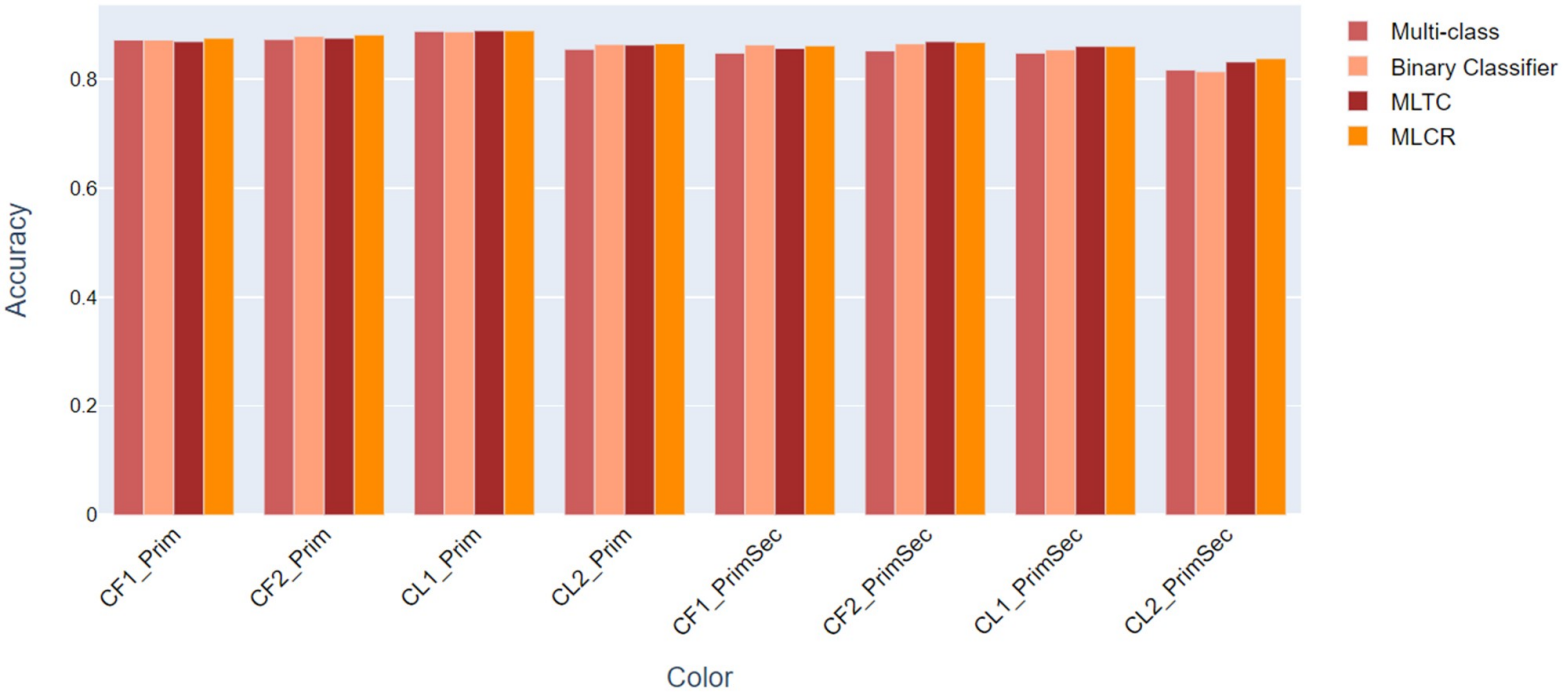
Overview of the performance of the various classifiers studied.

Fig 9 summarizes the performance of the various classifiers by means of bar graphs, with binary classification method 1 now excluded, indicating that all of the classifiers are comparable. However, in general the ensemble classifier using MLCR shows better performance than the multi-class classifiers and combined-binary classifiers.

## Discussion

It has been repeatedly demonstrated that color is a useful discriminative feature in image-based flower recognition [1, 33]. As photographic images of flowers are made under varying and usually non-optimal circumstances, color detection of flowers is a far from easy task and thus hard to automate. Often the color histogram has been taken as method of choice. In contrast, we have explored color labels, a choice motivated by the fact that color labels are commonly employed by taxonomists in describing flowers. Automated color detection based on color labels offers certain benefit to the taxonomist. When used as part of a computer-based system that is able to provide the name of a flower species in an image, color labels can be used as part of an explanation of why the flower is classified as a certain species. However, a color label is in itself insufficient as a feature for flower identification, as different flowers often have the same color, implying that color can not uniquely predict the name of a species. Therefore, **color features have to be combined with other morphological features**.

Even though the results from the classifiers are not significantly different, for the analysis, we only use the results from the ensemble classifier MLCR which slightly outperforms the other classifiers.

**Fig 10:**
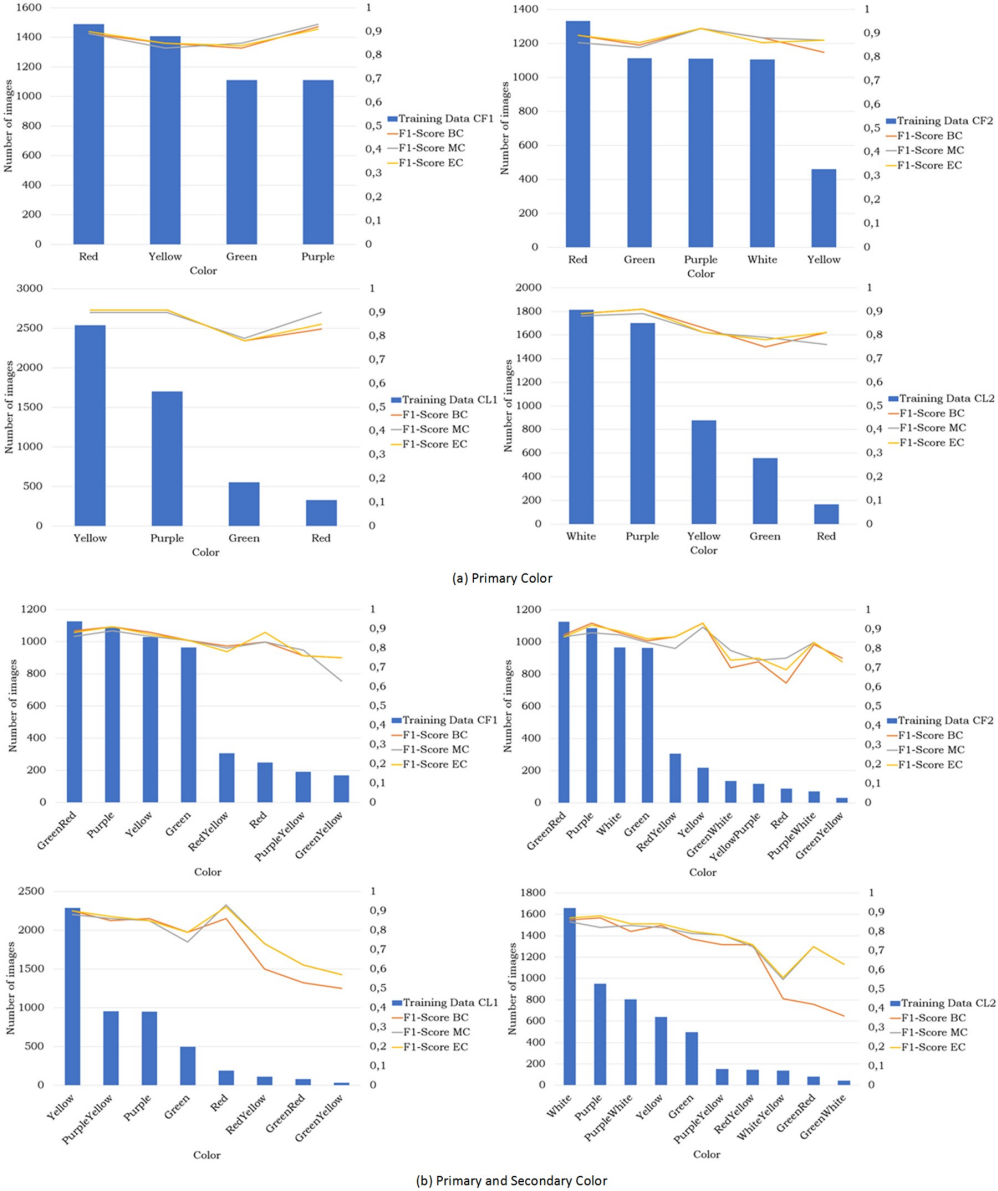
Number of images for each color related to the *F*_1_-score of the various classifiers.

As discussed in the previous paragraph, the dataset used in our research was very challenging and reflects common difficulties met in automatic flower identification in the real world. Fig 10 indicates that the dataset suffers from class imbalance; there is a big difference between the number of samples belonging to the majority and the minority class. However, as can be seen in Fig 10, the color classes that have a limited number of training samples do not always have a low *F*_1_-score, as for example illustrated by the primary color ‘Red’ for CL1 and CL2, and ‘PurpleWhite’, ‘GreenYellow’ for CF2, ‘GreenRed’, and ‘GreenWhite’ for CL2 using both primary and secondary color. One explanation is that **the class weights used to handle the imbalance in the data during training had indeed a positive effect on the performance for the minority class**. Another possibility is that images in **the minority class with a good *F*_1_-score have a similar appearance** compared to other minority classes so that the classifier can recognize them more easily.

**Fig 11:**
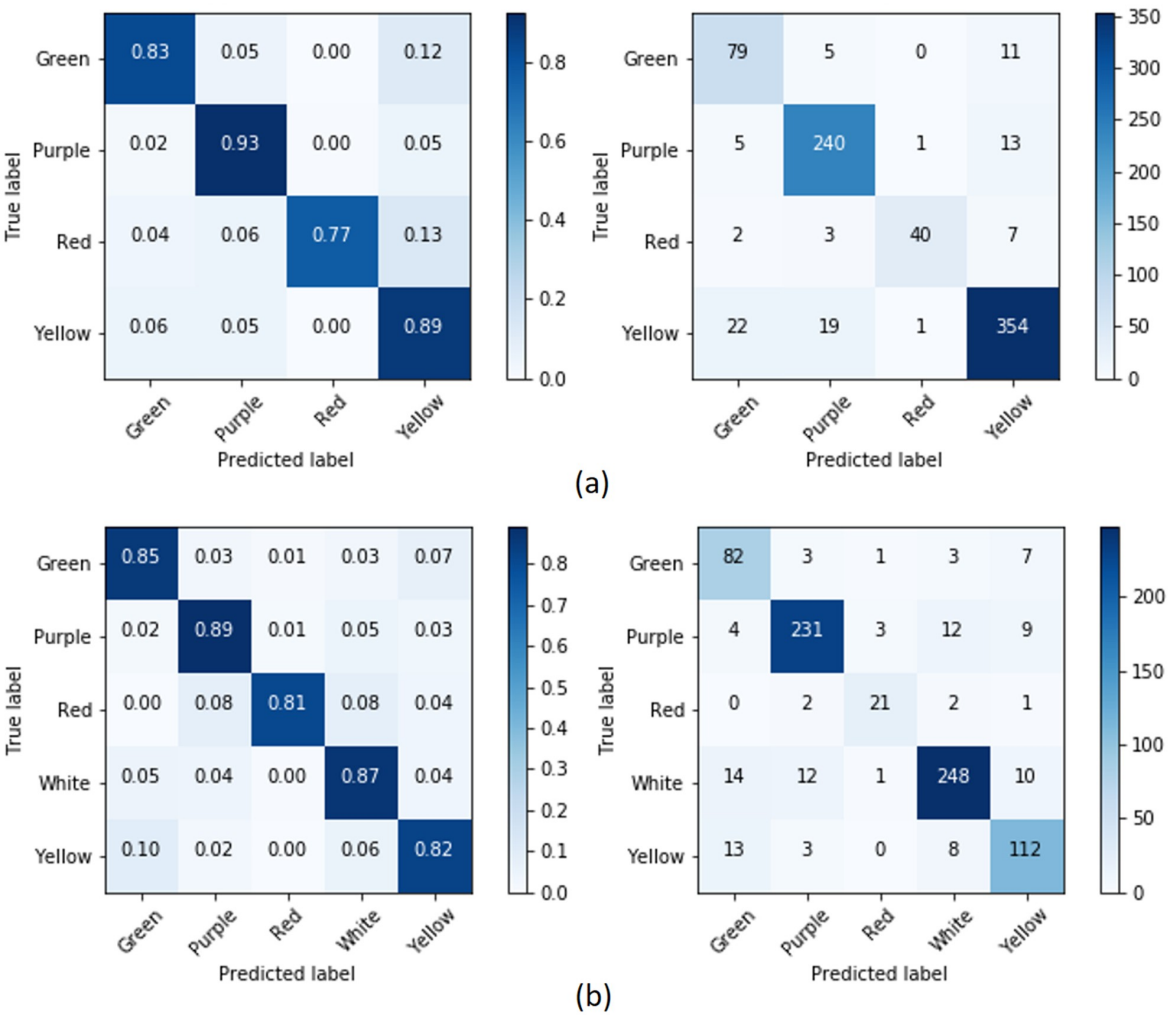
The confusion matrices for primary color: (a) CL1 and (b) CL2 using the ensemble classifier.

**Fig 12:**
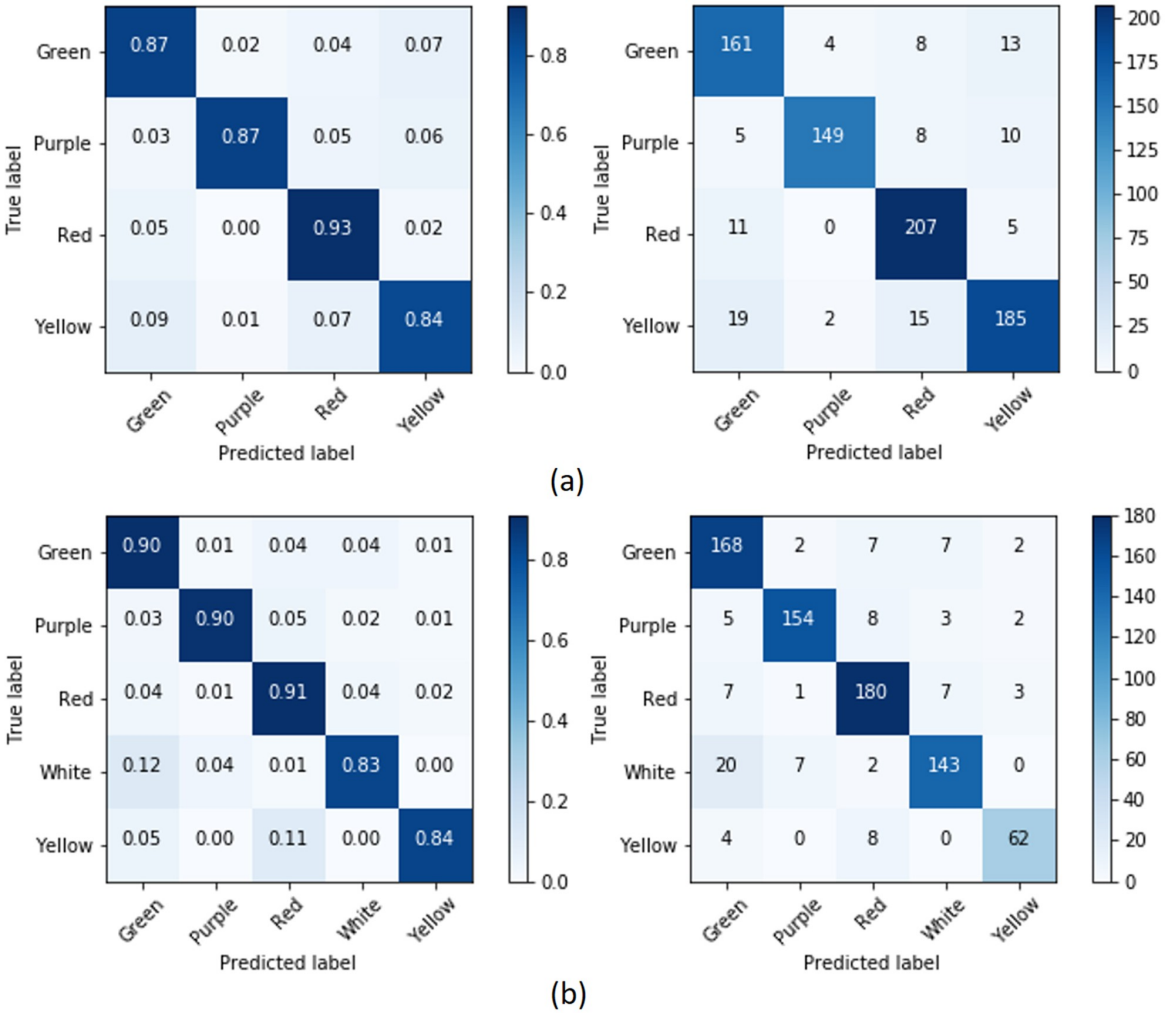
The confusion matrices for primary color: (a) CF1 and (b) CF2 using ensemble classifier.

Next, we consider the confusion matrices for each classifier to obtain more detailed information about which colors are hard to predict. **Our automated color detection system possesses a little bit of overfitting, but not too much**. We may note from the confusion matrices, for example from Fig 11, that the classes Yellow and Purple, which have the highest amount of training data, have high accuracy while the other classes are often classified into these classes. Nevertheless, the other classes can still achieve high accuracy (for primary color most of classifications are above 80%), even though there is a big difference in the amount of training data for the class values with the highest and the lowest number of samples (the ratio between the two is between 1.5–13). Using primary color of the labellum, with color schemes 1 and 2, ‘Red’ is the hardest color to predict. ‘Red’ is often predicted as ‘Yellow’ with color scheme 1 and ‘White’ with color scheme 2. Sometimes, it is also predicted as ‘Purple’ in both color schemes. In Fig 12, for the color of flower, ‘Yellow’ is the most difficult color to predict using color scheme 1, whereas it is ‘White’ for color scheme 2. For both color schemes, these colors are often predicted as ‘Green’. The other confusion matrices are provided in the supplement S1 Fig.

**Fig 13:**
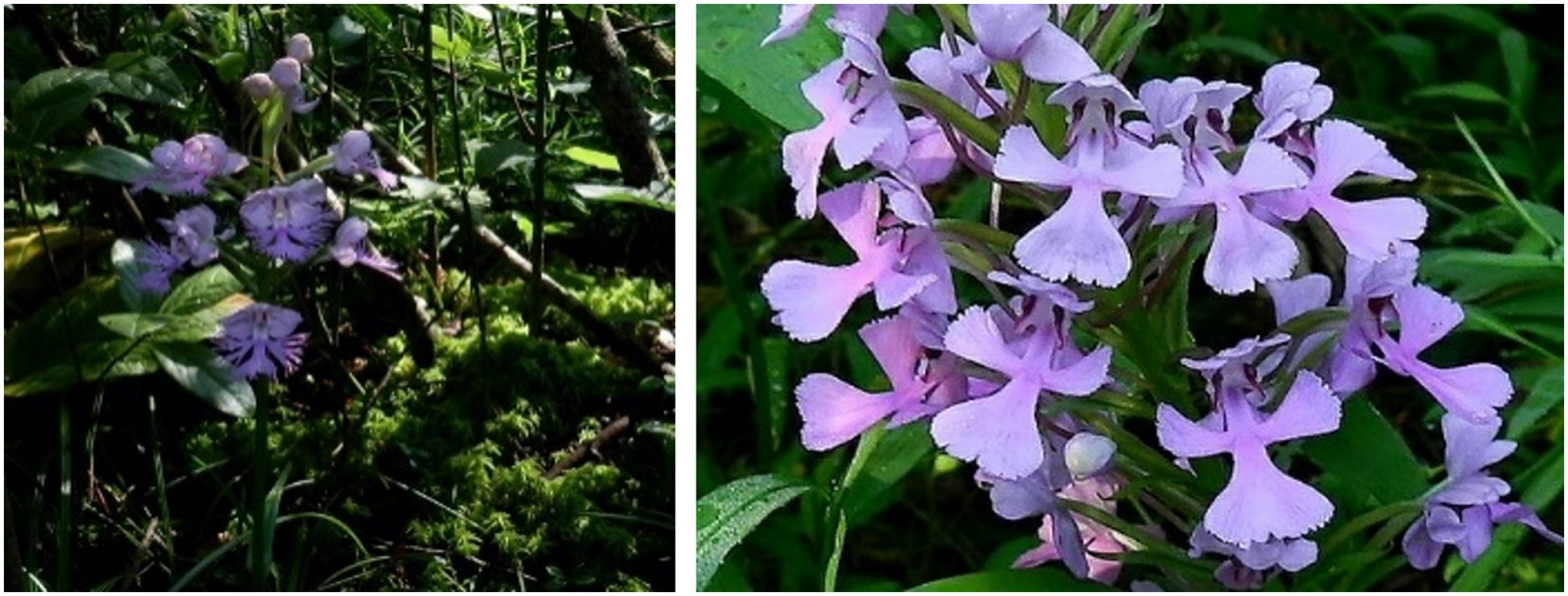
The color of the orchids seen in the pictures (purple) corresponds to the literature, whereas the classifier predicted white as their color.

**Fig 14:**
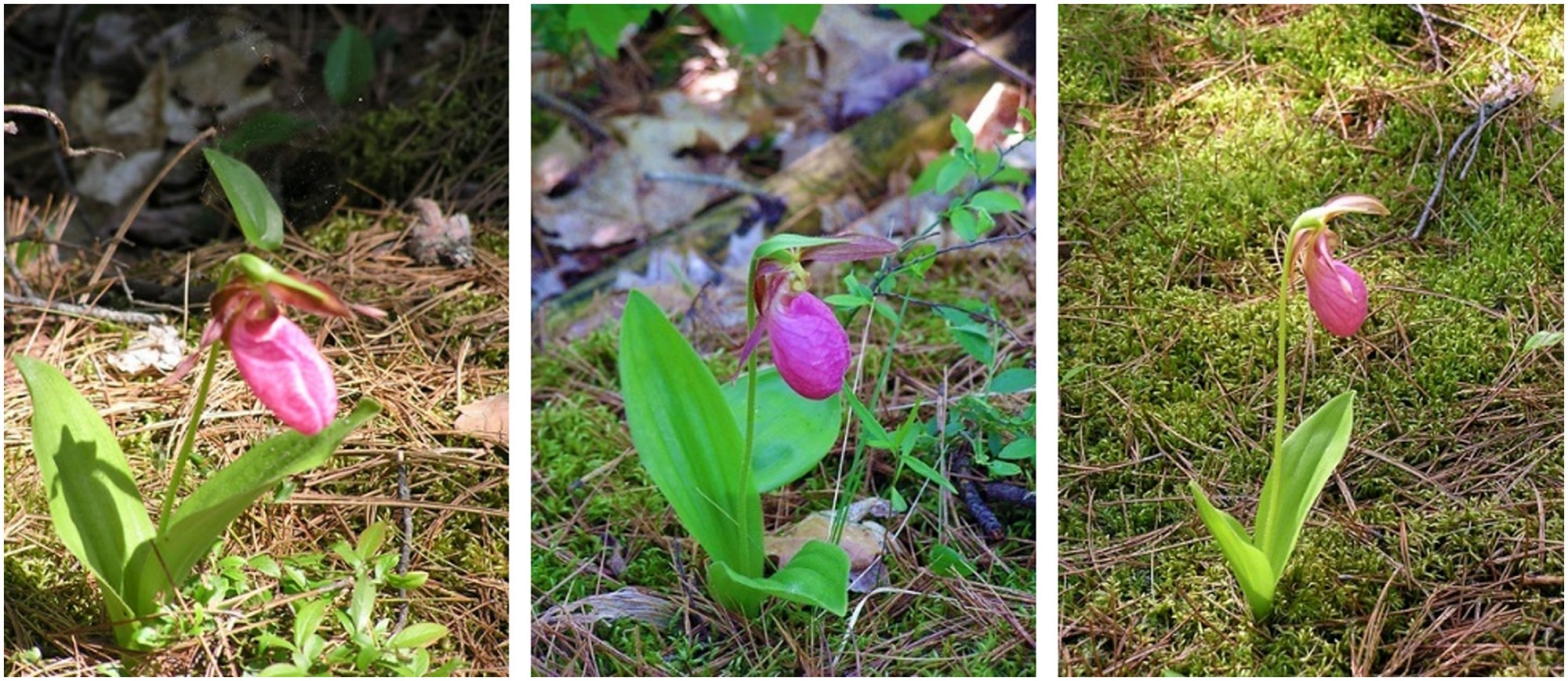
According to the literature, the orchid should be white. Yet, the picture clearly shows a pink-purple flower, where the classifier predicted purple as color (which includes pink in our color scheme), which was counted as being incorrect.

**Fig 15:**
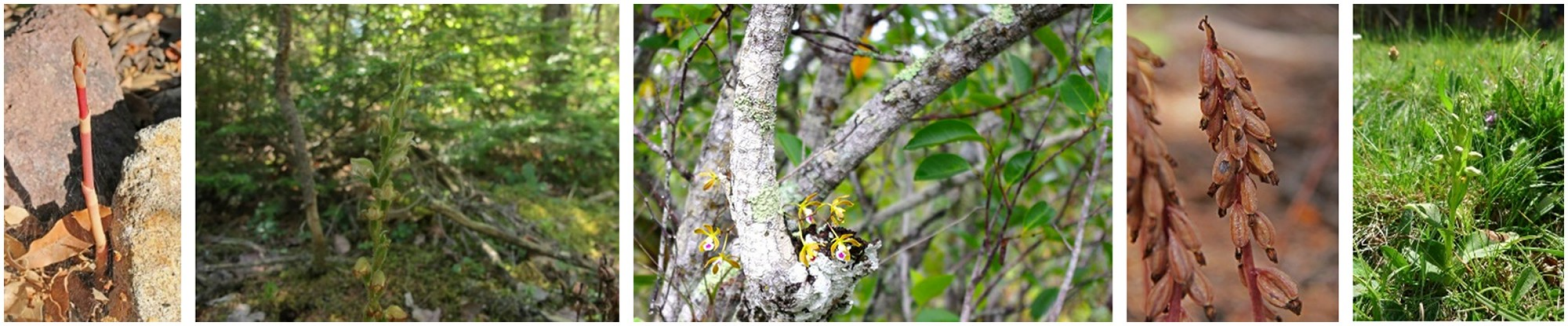
Examples of images that are hard to classify because of photographic imperfections or non-blooming stages of the orchid.

It is worthwhile to examine the images used in testing in more detail to understand why the classifiers often achieved a good performance and sometimes also failed. As a consequence, **the 20% images that were misclassified were further analyzed**. Three main reasons for the misclassification were uncovered:

1. It may be the case that the color of the flower in the image corresponds to that in the literature, whereas the predicted color is different. An example to illustrate this situation is shown in Fig 13. By their uncertain nature, all classifiers make sometimes mistakes.
2. The color appearance in the misclassified images sometimes differs from the colors mentioned in the literature. In that case, the predicted color may correspond either to the color mentioned in the literature (counted as correct), or to the color appearing in the image (which we count as incorrect). Fig 14 shows an example of a case where a seemingly correct prediction is counted as incorrect. Hence, in this case either the literature is mistaken, or the picture taken had for some reason colors that were not described previously. In both cases, a decision has to be made as whether the prediction is considered to be correct or incorrect. We decided to be conservative in our assessment.
3. An image often not only includes the flower that is to be classified, but also other flowers, pictured from different angles, and a variety of backgrounds such as grass, leaves, trunks, etc. There are also some images that show the orchid’s seed pods or flower buds which may have a color that differs from the blooming flower. These images were classified by us as **hard images** as it is almost impossible to detect the color correctly. Fig 15 gives some examples.

Not much can be done about the misclassified images of type (1) as our classifiers are already optimal. The distribution of the other two misclassified image types (2) and (3) is shown in Table 8, indicating that misclassification is reasonably balanced between the two categories.

**Table 8:** Distribution of misclassified images.

Color Scheme	Potentially Wrong Labels	Hard Images
**CF1 (100 images)**	32	29
**CF2 (95 images)**	19	24
**CL1 (89 images)**	35	22
**CL2 (108 images)**	26	23

Furthermore, we carried out an additional experiment, where we manually corrected the potentially wrong labels based on the literature and as sometimes an orchid was known to have a number of alternative colors, these colors were added. Next, we counted a prediction as being correct if the predicted color occurred among the colors mentioned in the database. Table 9 shows the accuracy before and after label adjustment using set-membership to count the correct predictions. As can be seen, the classifier’s performance improved between 1.5–4.5% after implementing these modifications.

**Table 9:** Effect on the accuracy of the classifier after label modification and counting correct predictions in terms of set-membership.

Color Scheme	Original Accuracy	Accuracy after Modification
**CF1**	87.5	91.5
**CF2**	88.2	90.5
**CL1**	88.9	93.2
**CL2**	86.5	89.8

### Commutativity of color

As mentioned in the material and methods section, we used commutativity of primary and secondary color to reduce the number of color combinations, hoping for an improvement in the classification performance. The reader may wonder whether this effect really occurred, which is why here attention is payed to this issue. We limited the study to the multi-class classification as computation times for the experiments would have increased considerably. This yielded 12, 15, 12, and 16 color combinations for CF1, CF2, CL1, and CL2, respectively. In Table 10, the performance of the multi-class classifier using these color combinations is compared to using color commutativity, confirming a general decrease in performance if commutativity of color is not deployed.

**Table 10:** The performance of the multi-class classifier using primary and secondary color after dropping the property of color commutativity.

Color Scheme	Accuracy	F_1_
**CF1**	0.837	0.756
**CF2**	0.825	0.711
**CL1**	0.830	0.688
**CL2**	0.810	0.675

## Conclusion and future work

The best classifier is able to detect the color of the flower and labellum based on their appearance in the image pretty well. Of course, applying segmentation to the images, thus isolating the orchid from the background, most likely offers better performance. However, it appears that our results are satisfactory when one wishes to detect orchid colors and segmentation is not needed. This offers the advantage that the user is not confronted with the burden of manual segmentation, whereas automated segmentation of pictures that include plants with their complex background may not be feasible. Even though there is still a little bit of overfitting, the classifier is able to suppress it. Reliable color detection of orchids based on color labels can be used as input to an automated image-based flower recognition program, which may include models that are able to provide a better explanation of the classification than deep learning is able to offer.

## Supporting information

S1 Fig Detailed figures. The schemas can be used to understand the proposed methods easily. The confusion matrices have acted as the basis for the computation of the various performance scores in the paper.
(PDF)
