# Supplementary material for: Automated color detection in orchids using color labels and deep learning
Source: PLoS One. 2021 Oct 27;16(10):e0259036. doi: 10.1371/journal.pone.0259036 (PMC8550396; doi:10.1371/journal.pone.0259036)
Supplement: S1 Fig — The schemas can be used to understand the proposed methods easily. The confusion matrices have acted as the basis for the computation of the various performance scores in the paper. (PDF) [file pone.0259036.s001.pdf]

S1 Fig. Detailed Figures

Fig 1 shows a schematic diagram of combined-binary classifiers using deep learning.

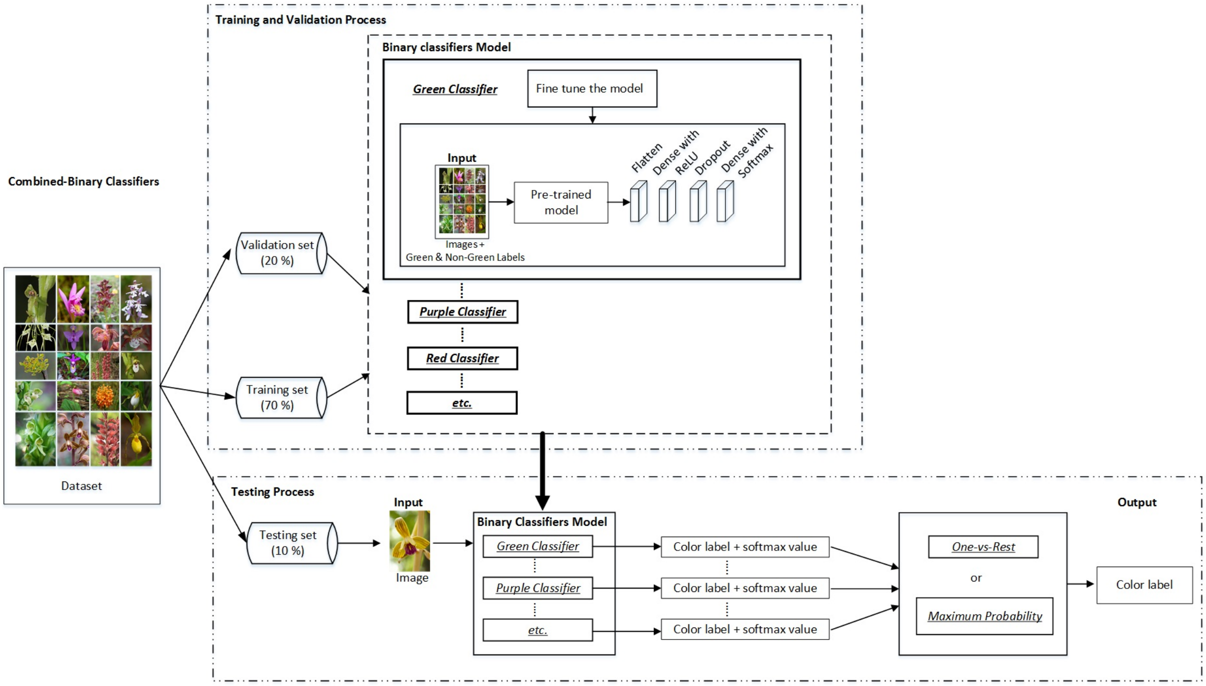

Fig 1: The scenario for the combined-binary classifier.

Fig 2 depicts a schematic diagram for the ensemble classifiers.

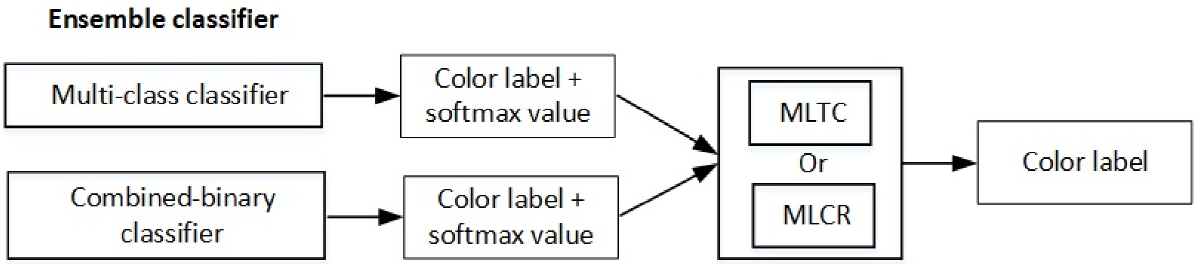

Fig 2: The scenario for the ensemble classifier.

The confusion matrix for each color schemes obtained by different classifiers on primary color is shown in Figs 3, 4, 5, 6, while for primary and secondary color together they are shown in Figs 7, 8, 9, 10.

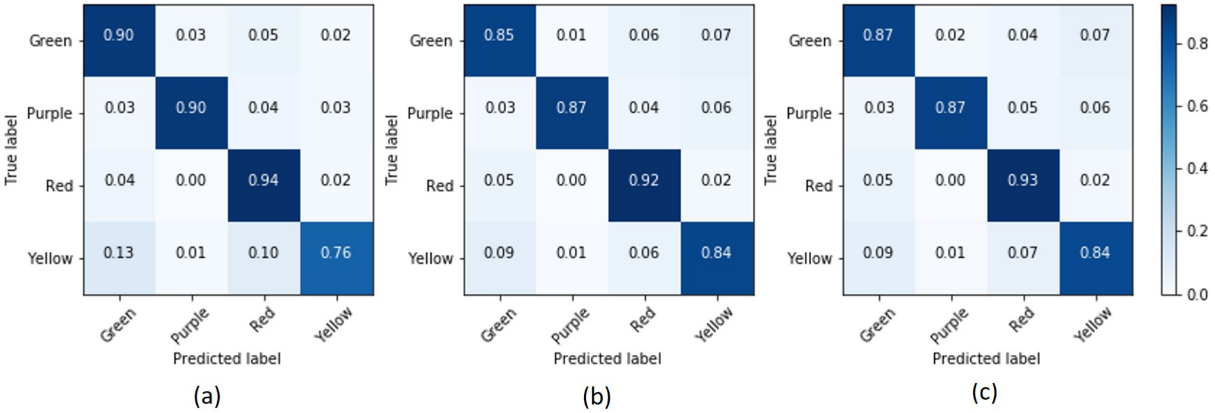

Fig 3: The confusion matrix of CF1 on primary color.
(a) Multi-class (b) Combined binary (c) MLCR Ensemble.

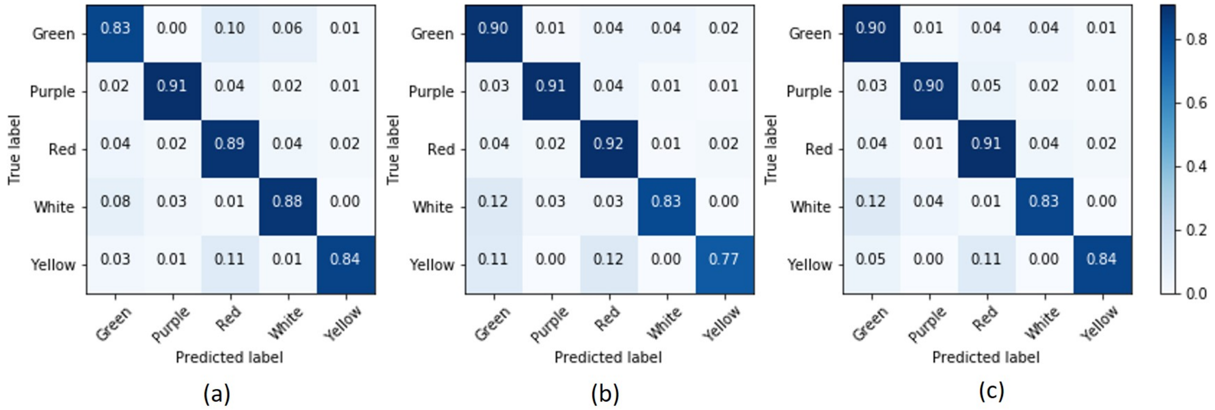

Fig 4: The confusion matrix of CF2 on primary color.
(a) Multi-class (b) Combined binary (c) MLCR Ensemble

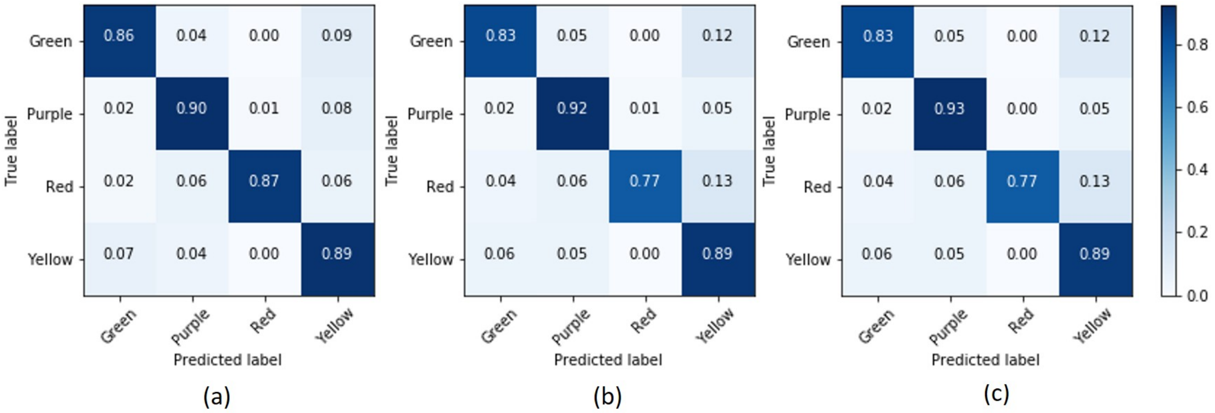

Fig 5: The confusion matrix of CL1 on primary color.

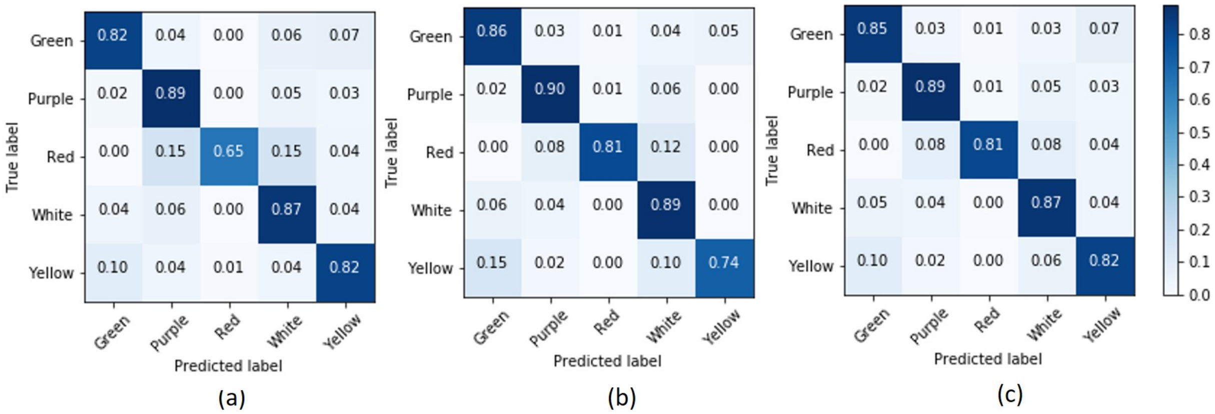

Fig 6: The confusion matrix of CL2 on primary color.

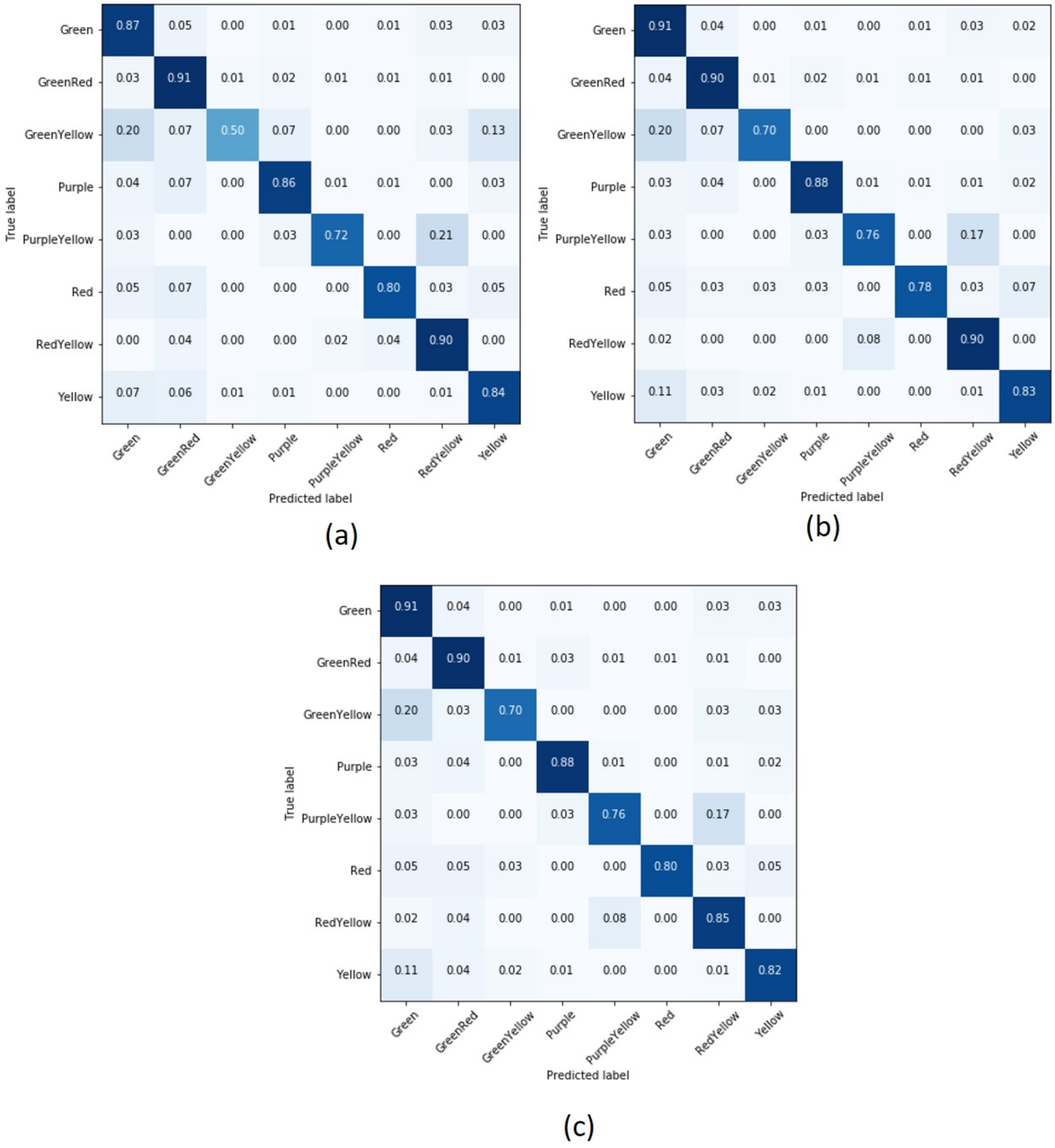

Fig 7: The confusion matrix of CF1 on primary and secondary color.

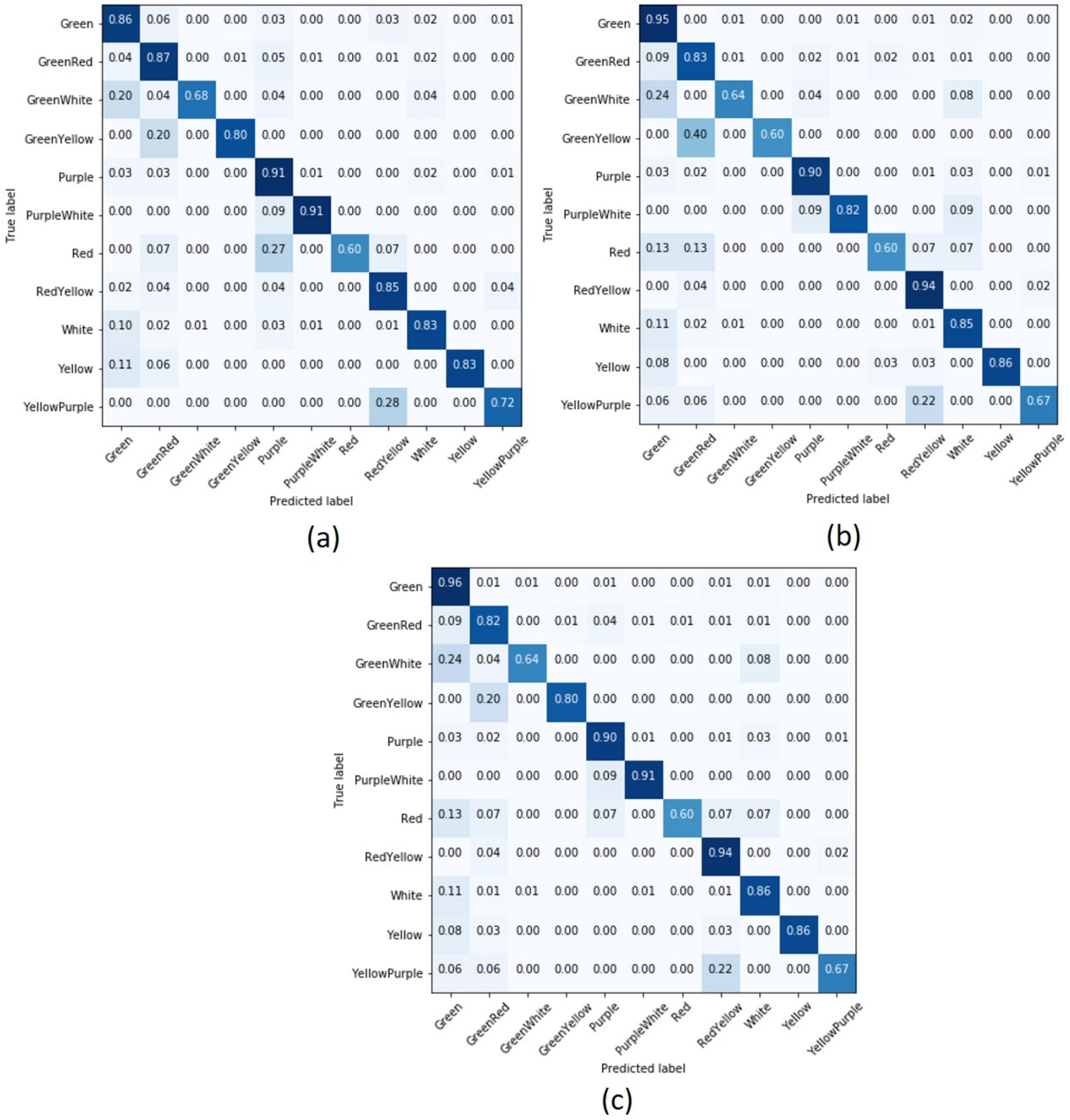

Fig 8: The confusion matrix of CF2 on primary and secondary color.

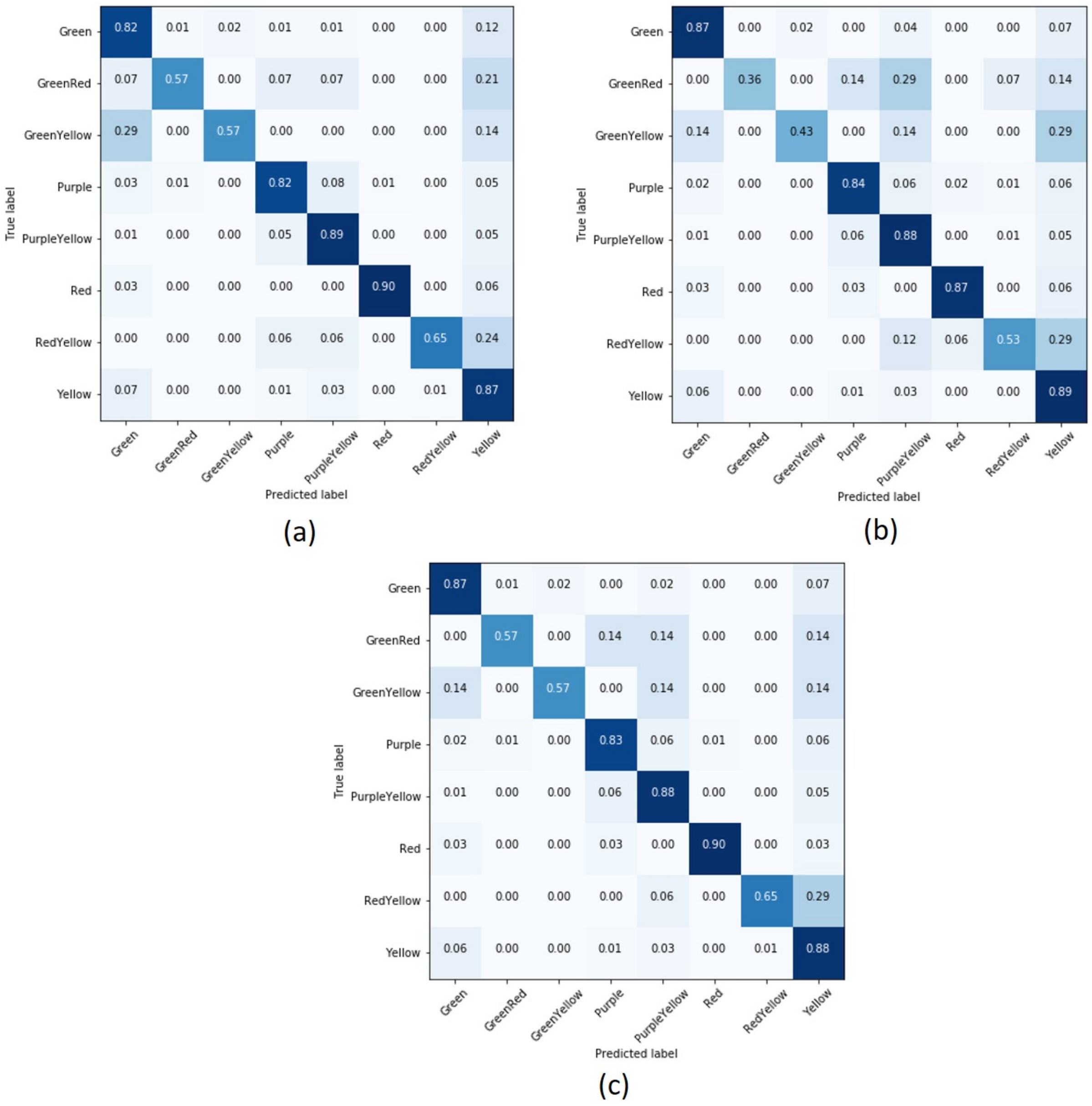

Fig 9: The confusion matrix of CL1 on primary and secondary color.

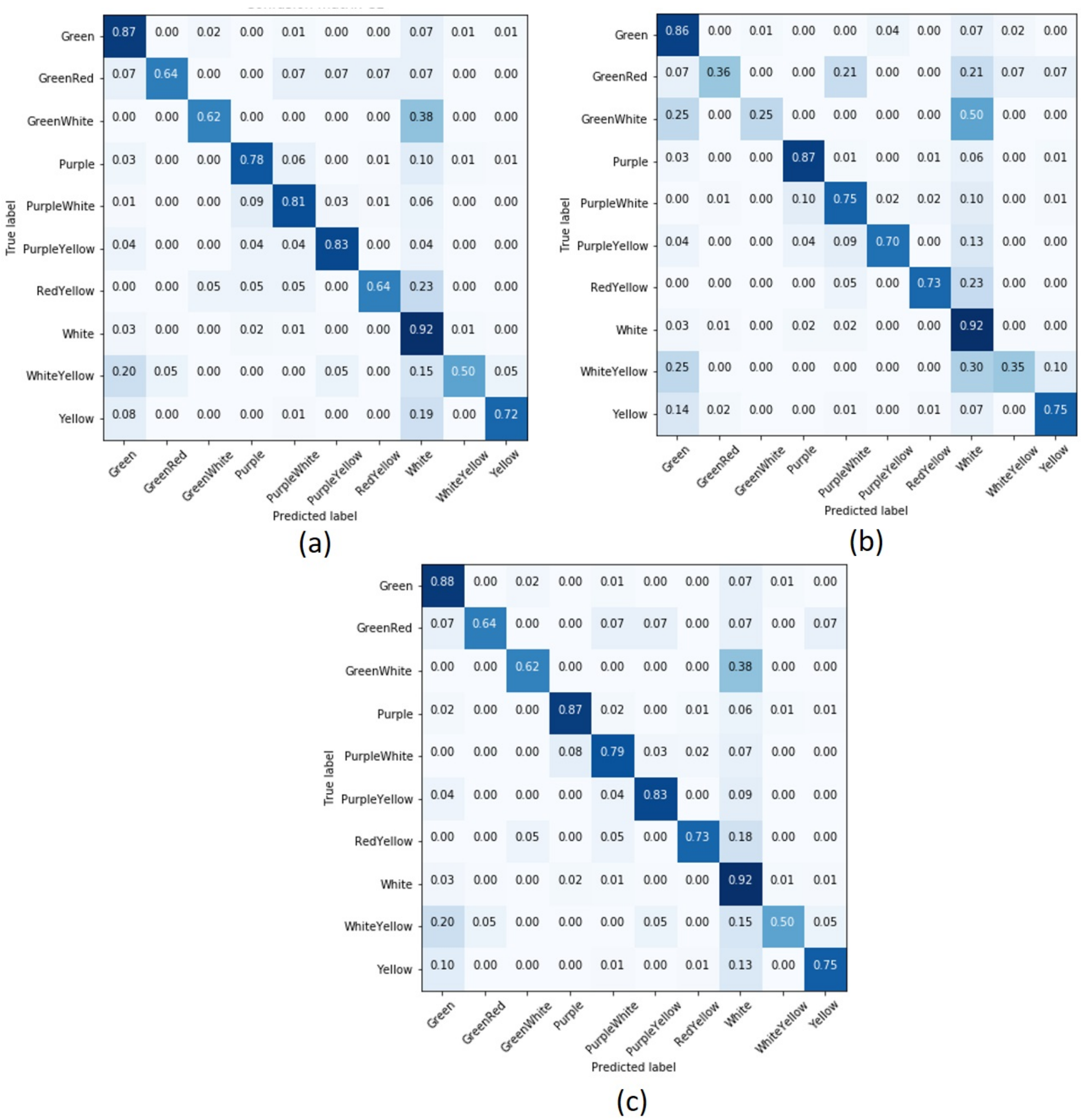

Fig 10: The confusion matrix of CL2 on primary and secondary color.
